# Lnc AC016727.1/BACH1/HIF-1 α signal loop promotes the progression of non-small cell lung cancer

**DOI:** 10.1186/s13046-023-02875-y

**Published:** 2023-11-10

**Authors:** Li Zhang, Jingtian Liang, Hao Qin, Yin Lv, Xiucheng Liu, Zhuoqun Li, Zhixiang Chao, Caili Jia, Xichun Qin, Hao Zhang

**Affiliations:** 1 Thoracic Surgery Laboratory, Xuzhou Medical University, Xuzhou, 221006 Jiangsu Province China; 2 Department of Thoracic Surgery, Affiliated Hospital of Xuzhou Medical University, 99 West Huaihai Road, Xuzhou, 221006 Jiangsu Province China

**Keywords:** lncRNA AC016727.1, miR-98-5p, Aerobic glycolysis, BTB and CNC homolog 1, Hypoxia, Non-small cell lung cancer

## Abstract

**Background:**

Long noncoding RNAs (lncRNAs) have been reported to play vital roles in the development and progression of cancer. However, their biological significance and functional mechanisms in non-small cell lung cancer (NSCLC) are mostly unclear.

**Methods:**

We performed RNA-sequencing to predict the differential expression of lncRNAs in clinical NSCLC and paired paracancerous lung tissues. To identify lncRNA expression, quantitative polymerase chain reaction (qPCR) was used. Using both cell and mouse models, We studied lncRNA AC016727.1’s function in NSCLC growth and metastasis. Western blot assays, dual luciferase reporter assays, and chromatin immunoprecipitation were used to analyze the functional mechanism of lncRNA AC016727.1.

**Results:**

Our larger NSCLC cohorts validated that the lncRNA AC016727.1 was upregulated in 94 paired NSCLC tissues and correlated with poor survival. Functionally, lncRNA AC016727.1 downregulation inhibited NSCLC cell proliferation, aerobic glycolysis, EMT, and migration, inducing apoptosis. Conversely, upregulated lncRNA AC016727.1 expression exhibited the opposite effect, promoting NSCLC cell survival. Importantly, lncRNA AC016727.1 knockdown inhibited lung cancer growth and slowed the progression of lung metastasis in nude mouse models. Mechanistically, lncRNA AC016727.1 upregulated *BACH1* target gene expression by acting as a sponge for miR-98-5p, thereby functioning as a competing endogenous RNA. The function of lncRNA AC016727.1 is mediated by the miR-98-5p/BACH1 axis in NSCLC cells. Meanwhile, the transcription factor HIF-1α can bind to the promoter and activate lncRNA AC016727.1 transcription. lncRNA AC016727.1 regulates HIF-1α expression via BACH1 in NSCLC and forms the lncRNA AC016727.1/BACH1/HIF-1α signaling loop under hypoxic conditions.

**Conclusion:**

Our study reveals a novel lncRNA AC016727.1/BACH1/HIF-1α signaling loop in the progression of NSCLC under hypoxic conditions, suggesting that lncRNA AC016727.1 could act as a useful biomarker for NSCLC and a new therapeutic target.

**Supplementary Information:**

The online version contains supplementary material available at 10.1186/s13046-023-02875-y.

## Background

Lung cancer, a malignant bronchial mucosa or gland tumor, is one of the most prevalent cancers globally, with 2.2 million cases emerging and 1.79 million fatalities reported annually [1]. Lung cancer has two broad histological subtypes: non-small cell lung cancer (NSCLC,85%), which includes large-cell carcinoma, squamous-cell carcinoma, as well as adenocarcinoma and small-cell lung cancer (15%) [2]. The most prevalent subtype of NSCLC is adenocarcinoma [3], followed by squamous cell carcinoma [4]. While surgery remains the primary treatment for early-stage NSCLC [5], patients with advanced lung cancer are typically ineligible for surgical intervention. Thus, research focused on the molecular mechanisms of lung cancer progression, aiming to identify candidate biomarkers and targets for therapy, is crucial for early detection and treatment.

Long noncoding RNAs (lncRNAs) are a class of non-protein-coding RNA transcript that participates in a variety of processes, both physiological and pathological, through epigenetic modulation and related signal transduction [6–9]. NSCLC pathogenesis is often derived from various complex genetic and epigenetic alterations [10]. lncRNAs have emerged as novel regulators with diverse functions and mechanisms that influence cancer progression [11]. In our study, we identified a novel upregulated lncRNA, lncRNA AC016727.1, in NSCLC through RNA sequencing analysis. However, the biological implications and molecular mechanisms of lncRNA AC016727.1 in NSCLC are currently unknown. Increasing evidence reveals that lncRNAs can compete with endogenous RNAs (ceRNAs) to regulate microRNA (miRNA) expression and their biological functions. For instance, LINC01123, which activates c-Myc by serving as a ceRNA for miR-199a-5p, promotes NSCLC progression [12]. Furthermore, As a ceRNA for miR-33a-5p, lncRNA JPX regulates Twist1 and the Wnt/β-catenin signaling pathway, consequently regulating carcinogenesis and metastasis in lung cancer [13].

A hallmark feature of the tumor microenvironment is hypoxia, which promotes tumor plasticity and heterogeneity, leading to an aggressive and metastatic tumor phenotype. In this process, activated hypoxia-inducible factor-1α (HIF-1α) plays a pivotal role in the adaptive response of tumor cells to changes in oxygen levels. HIF-1α activates downstream genes that are critical for tumor survival and progression at the transcriptional level [14]. It is well-documented that hypoxic conditions have a significant effect on lncRNA expression, with hypoxia-responsive lncRNAs contributing to tumorigenesis and tumor progression. For instance, HIF-1α antisense lncRNA is essential for maintaining and enhancing HIF-1α-mediated trans-activation and glycolysis [15], whereas lncRNA-AC020978 promotes proliferation and glycolytic metabolism by regulating the PKM2/HIF-1α axis in NSCLC [16]. However, whether lncRNA AC016727.1 is involved in molecular responses to hypoxic conditions remains unknown.

Thus, we mined the potential clinical relevance of lncRNA AC016727.1 in our cohorts of patients with NSCLC using transcript resequencing. We observed that the lncRNA AC016727.1 expression level was substantially elevated in tumor tissues and increased with tumor node metastasis (TNM) staging progression. Subsequent experiments indicated that lncRNA AC016727.1 is regulated by HIF-1α in five different NSCLC cell lines, resulting in the formation of a lncRNA AC016727.1/BACH1/HIF-1α signaling loop that influences NSCLC cell proliferation, migration, and invasion under hypoxic conditions. Furthermore, we evaluated the effect of the lncRNA AC016727.1/BACH1/HIF-1α signaling loop using mouse models at the individual level. Our findings suggest that lncRNA AC016727.1 is a potential therapeutic target and biomarker in NSCLC.

## Methods

### Clinical samples and RNA sequencing

Between June 2017 and October 2017, tissue specimens were collected from patients with NSCLC at the Affiliated Hospital of Xuzhou Medical University (Xuzhou, China). Tumor and paraneoplastic tissue samples were rapidly frozen in liquid nitrogen upon acquisition and preserved at − 80°C. Table 1 shows the clinicopathological features of patients with NSCLC. Table 2 shows the univariate and multivariate Cox regression analyses of the clinicopathological characteristics of patients with NSCLC.

**Table 1:** Correlation of the expression of Lnc AC016727.1 in NSCLC with clinicopathologic features

Characteristics	Lnc AC016727.1			*p*value
	Low(*N* = 52)	High(*N* = 42)	Total(*N* = 94)	
Age				0.18
≥ 55	29(30.85%)	30(31.91%)	59(62.77%)	
< 55	23(24.47%)	12(12.77%)	35(37.23%)	
Gender				0.74
Female	35(37.23%)	26(27.66%)	61(64.89%)	
Male	17(18.09%)	16(17.02%)	33(35.11%)	
Cancer type				0.19
LUAD	47(50.00%)	33(35.11%)	80(85.11%)	
LUSC	5(5.32%)	9(9.57%)	14(14.89%)	
Tumor size				0.002**
≥ 2 cm	16(17.02%)	27(28.72%)	43(45.74%)	
< 2 cm	36(38.30%)	15(15.96%)	51(54.26%)	
Tumor multiplicity				1
Multiple	12(12.77%)	10(10.64%)	22(23.40%)	
Single	40(42.55%)	32(34.04%)	72(76.60%)	
TNM stage				0.008**
I	32(34.04%)	13(13.83%)	45(47.87%)	
II	13(13.83%)	15(15.96%)	28(29.79%)	
III	7(7.45%)	14(14.89%)	21(22.34%)	
T stage				0.008**
T1	37(39.36%)	16(17.02%)	53(56.38%)	
T2	10(10.64%)	14(14.89%)	24(25.53%)	
T3	5(5.32%)	10(10.64%)	15(15.96%)	
T4	0	2(2.13%)	2(2.13%)	
Lymph node metastasis				0.03*
N0	31(32.98%)	17(18.09%)	48(51.06%)	
N1	14(14.89%)	10(10.64%)	24(25.53%)	
N2	4(4.26%)	13(13.83%)	17(18.09%)	
N3	3(3.19%)	2(2.13%)	5(5.32%)	
Distant metastasis				
M0	52(55.32%)	42(44.68%)	94(100.00%)	
M1		0	0	0
Mortality				0.001**
Survive	41(43.62%)	15(15.96%)	56(59.57%)	
Die	11(11.70%)	27(28.72%)	38(40.43%)	

^*^p < 0.05

^**^p < 0.01

**Table 2:** Univariate and multivariate Cox regression analysis for clinicopathological features associated with prognosis of 94 NSCLC patients

Features	Univariate analysis		Multivariate analysis	
	Hazard Ratio(95%CI)	*p*-value	Hazard Ratio(95%CI)	*p*-value
Gender (male vs. female)	2.08(1.09–3.94)	0.35	1.18(0.48–2.92)	0.72
Age (≥ 55 vs. < 55)	0.13(0.04–0.42)	0.18	0.21(0.06–0.73)	0.08
Tumor size(≥ 2 vs. < 2)	0.17(0.07–0.38)	0.25	1.929 (1.140–3.013)	0.06
TNM stage (I–II vs. III–IV)	0.61(0.27–1.353)	0.006**	0.37(0.14–0.95)	0.04*
T stage (T1–2 vs. T3–4)	3.31(1.63–6.68)	0.001**	0.30(0.08–1.15)	0.08
Lymph node metastasis (N0 vs. N1–3)	3.06(1.43–6.541)	0.004**	2.97(1.39–6.38)	0.05
Tumor multiplicity(Single vs. Multiple)	2.53(1.24–5.12)	0.06	0.88(0.35–2.20)	0.78
Cancer type(LUAD vs. LUSC)	1.18(0.55–2.53)	0.668	0.58(0.26–1.31)	0.19
Lnc AC016727.1 (low vs. high)	3.65(1.808–7.37)	0.013*	2.99(1.37–6.55)	0.03*

^*^P < 0.05

^**^P < 0.01

The Illumina HiSeq platform (Shanghai, China) was used for RNA high-throughput sequencing. Briefly, total RNA was depleted of rRNA using the NEBNext rRNA Depletion Kit (New England Biolabs, Inc., Massachusetts, USA) according to the manufacturer's recommendations. The NEBNext® UltraTM II Directional RNA Library Prep Kit (New England Biolabs) was used to construct the RNA libraries, which were assembled according to the manufacturer's recommendations. Using the BioAnalyzer 2100 (Agilent Technologies, Inc., USA) for quality control and library quantification. Library sequencing was conducted on the Illumina Hiseq instrument with 150 bp paired-end reads.

### Cell lines and culture conditions

We obtained six human NSCLC cell lines, namely A549, H1299, H292, H23, H1703, and H226, along with a BEAS-2B human normal lung epithelial cell line from Procell (Wuhan, China). In Dulbecco's modified Eagle medium with 10% fetal bovine serum, BEAS-2B and A549 cells were cultured. We cultured H1299, H292, H23, H1703, and H226 cells in Roswell Park Memorial Institute-1640 containing 10% fetal bovine serum. A 5% CO_2_ cell culture incubator maintained all cell lines at 37 °C. The medium was obtained from KeyGEN BioTECH (Jiangsu, China), and 10% fetal bovine serum was sourced from Clark (Australia). All cells were confirmed to be free of mycoplasma contamination. Cells were cultured in a hypoxia chamber with 1% O_2_ balanced with CO_2_ and nitrogen for hypoxic treatment. (China Innovation Instrument Co., Ltd.)

### Total RNAoifd extraction and real-time quantitative polymerase chain reaction (RT-qPCR)

RNA extraction was performed using Trizol (Invitrogen, Carlsbad, CA, USA), whereas cDNA synthesis was carried out using the HiScript First Strand cDNA Synthesis Kit (Nanjing Vizimax Biotechnology, Nanjing, China). The UltraSYBR One-Step RT-qPCR Kit (CWBIO, Beijing, China) was used for the RT-qPCR analysis, which was performed in accordance with the manufacturer's instructions. We used the miRNA extraction kit (TIANGEN, Beijing, China) and the plus-tail miRNA synthesis kit (TIANGEN) following the manufacturer's instructions to measure miRNA levels. Endogenous regulation was provided by U6 snRNA. Additional file: Table S1 presents the primer sequences utilized in this study.

### Immunohistochemistry

Immunohistochemical (IHC) staining for Ki67 was performed on paraffin-embedded tumor tissue sections following experimental instructions. Final images were captured using the Pannoramic MIDI system.

### Preparation of lentiviral transfections and stable cell lines

GeneChem Co. designed and synthesized the overexpression (OE) lncRNA AC016727.1 lentivirus constructs and short hairpin RNAs (shRNAs). Using Lipofectamine 2000 transfection reagent (Invitrogen), shRNA-lncRNA AC016727.1 and shRNA-control were transfected into A549, H1299, and H23 cell lines. For H1703 and H226 cell lines, empty vector and OE-lncRNA AC016727.1 were used. Before being exposed to 2 ng/mL puromycin for 2 weeks with medium changes every three days, cells were infected with lentivirus for 48 h.

Lipofectamine 2000 transfection reagent (Invitrogen) was used to transfect Si-BACH1 into A549 and H1299 cell lines. Six hours after transfection, the fresh medium replaced the old, and experiments were completed within 96 h.

GeneChem Co. designed and synthesized plasmids overexpressing BACH1. Lipofectamine 2000 (Invitrogen) transfected A549 and H1299 cells with BACH1. Six hours after transfection, a fresh medium was used to replace the old medium, and experiments were completed within 96 h.

### Animal models

Changzhou Cavens Laboratory Animal Ltd (Jiangsu, China) provided the Balb/c nude mice (4–6 weeks old). Animal Protection and Utilization Committee of Xuzhou Medical University (Xuzhou, China) approved all animal experiments. To assess in vivo cell growth and metastatic potential, lung metastasis and subcutaneous xenograft models were developed. A549 cells transfected with either sh-control or sh-lncRNA AC016727.1 were used in animal experiments. Transfected A549 cells (1 × 10^5^ cells per injection) were subcutaneously injected into the right flank of mice for the subcutaneous xenograft model. Tumor size was assessed every 7 days, and after 4 weeks, the tumors were excised for volumetric assessment. Transfected A549 cells were injected into the tail vein as part of the lung metastasis model (1 × 10^5^ cells/dose). After 6 weeks, the mice were euthanized, and lung metastases were identified by hematoxylin and eosin (H&E) staining. Additionally, in C57BL/6 mice, an orthotopic lung tumor model was developed. Sodium pentobarbital (60 mg/kg) was injected intraperitoneally to anesthetize these mice. Following adequate anesthesia, luciferase-labeled Lewis lung carcinoma (LLC) (1.0 × 10^6^ cells) mixed with 50% Matrigel matrix (Corning Inc., Corning, NY, USA) was orthotopically injected into the left lung.

### Western blot analysis

The radioimmunoprecipitation assay lysis solution was used to lyse the cells on ice for 30 min, and the cells were then centrifuged at 15,000 xg for 30 min at 4 °C. Protein samples were examined using western blotting after cell extracts were boiled at 100 °C for 5 min. Polyacrylamide gel electrophoresis with sodium dodecyl sulfate was used to separate proteins, followed by transferring them to 0.45-μm polyvinylidene fluoride membranes (MilliporeSigma, Burlington, MA, USA). The membranes were subjected to blocking with a 5% skim milk solution for a duration of 1 h. Following this, the membranes were incubated at 4 °C with primary antibodies against Snail2/Slug, N-cadherin, vimentin, E-cadherin, BACH1, hexokinase II (HK2), phosphofructokinase-2 (PFK2), monocarboxylate transporter protein 1 (MCT1), HIF1α, and β-tubulin. After that, the membranes were treated for 1 h at room temperature with corresponding secondary antibodies. Bands of immunoreactive protein were visualized. Additional file: Table S2 lists the antibodies used in this study.

### Cell proliferation assay

The 5-ethyl-2' deoxyuridine (EdU) and cell Counting Kit-8 (CCK-8) assays were used for assessing cell proliferation. In 96-well plates, cells (3 × 10^3^) were seeded, and at 24, 48, and 72 h, the recommended volume of CCK-8 solution (Vicmed, Jiangsu, China) was added. EdU binding assays were conducted using the EdU binding kit according to the directions. In brief, cells (3 × 10^3^) were seeded in 96-well plates and treated with 50 µM EdU for 2 h. The incorporation of EdU was assayed after cells were fixed and stained with Apollo dye. Finally, Hoechst 33342 was used to stain cell nuclei.

### Colony formation assay

In petri dishes, 500 cells were plated, and they were incubated at 37 °C in a humid environment with 5% CO_2_. The following steps involved washing the colonies with phosphate-buffered saline, fixing them in 4% paraformaldehyde for 20 min, and staining them for 30 min with 0.5% crystal violet. The Pannoramic MIDI system was used to capture photographs.

### Cell invasion and migration assay

Collected cells were suspended in a serum-free medium. After seeding 1 × 10^5^ cells into Matrigel chambers (BD Bioscience, USA), they were incubated at 37 °C for 24 h. After that, the chambers were transferred to 24-well plates with 20% serum. Cells adhering to the lower membrane surface were fixed with 4% paraformaldehyde and stained with 0.1% crystal violet after 24 h, whereas those still on the upper membrane surface were removed using a cotton swab. After that, cells were counted using an optical microscope.

### Luciferase reporter analysis

The 3′-untranslated region (UTR) of lncRNA AC016727.1 or BACH1's wild-type (wt) or mutant (mut) sequences were cloned into the pGL3 vector (Promega, Madison, WI, USA), respectively. Renilla luciferase reporter plasmids with wt or mut of the 3′-UTR of lncRNA AC016727.1 or BACH1, along with miRNA, were co-transfected into 293 T cells. The pGL3 vector and the pGL3-lncRNA AC016727 were cloned with the lncRNA AC016727.1 promoter containing various hypoxia response elements (HREs). Under hypoxic conditions, the pGL3-lncRNA AC016727.1 vector was co-transfected with si-HIF-1ɑ or si-control. A dual luciferase reporter assay system (firefly luciferase and Renilla luciferase) (Promega) was used to quantify luciferase activity 24 h after transfection, following the manufacturer's instructions.

### Apoptosis analysis

The annexin V fluorescein isothiocyanate/propidium iodide apoptosis assay kit (Kogan Biotechnology, Nanjing, China) was used to analyze apoptosis following the manufacturer's recommendations.

### Determination of Extracellular acidification rate (ECAR) and Cellular oxygen consumption rate (OCR)

The Seahorse XFe 96 Extracellular Flux Analyzer (Seahorse Bioscience) was used to determine ECAR and OCR. Experiments were performed following the manufacturer's instructions. The Seahorse XF Glycolytic Stress Test Kit and the Seahorse XF Cell Mitochondrial Stress Test Kit were used to assess ECAR and OCR, respectively. Seahorse XF 96 cell culture microplates were seeded with 1 × 10^4^ cells per well. Following baseline measurements, 2-DG (a glycolysis inhibitor), oligomycin (an inhibitor of oxidative phosphorylation), and glucose were injected into each well at the designated time intervals for ECAR (indicated as mpH/min). OCR (indicated as pmols/min) was detected using antimycin A (a mitochondrial complex III inhibitor), p-trifluoromethoxycarbonyl cyanophenylhydrazone (an oxidative phosphorylation reversible inhibitor), rotenone plus (a mitochondrial complex I inhibitor), and oligomycin.

### Chromatin immunoprecipitation (ChIP)

To investigate the intracellular interaction between HIF1 and the HRE of the lncRNA AC016727.1 promoter, ChIP tests were performed using a ChIP kit (beyotime, China). Cells were formaldehyde-crosslinked and sonicated to 200–1000 bp (average length). The anti-HIF-1ɑ antibody (Proteintech) or IgG control was then used for immunoprecipitation. qRT-PCR was conducted to amplify the precipitated DNA using the primers described in Additional file 1: S1 Table.

### Statistical analyses

Data from the study are expressed as means ± standard deviations from at least three independent replications. GraphPad Prism 8.0.1 (GraphPad Software Inc., San Diego, CA, USA) was used for statistical analyses. The Chi-squared test was used to assess the link between JPX expression and clinical features of patients with NSCLC. The t-test was used to compare data between two groups, whereas one-way analysis of variance was used to compare data among three or more groups. Statistical significance was defined as a *p*-value < 0.05.

## Results

### Differential expression and clinical significance of lncRNA AC016727.1 in NSCLC

**Fig. 1:**
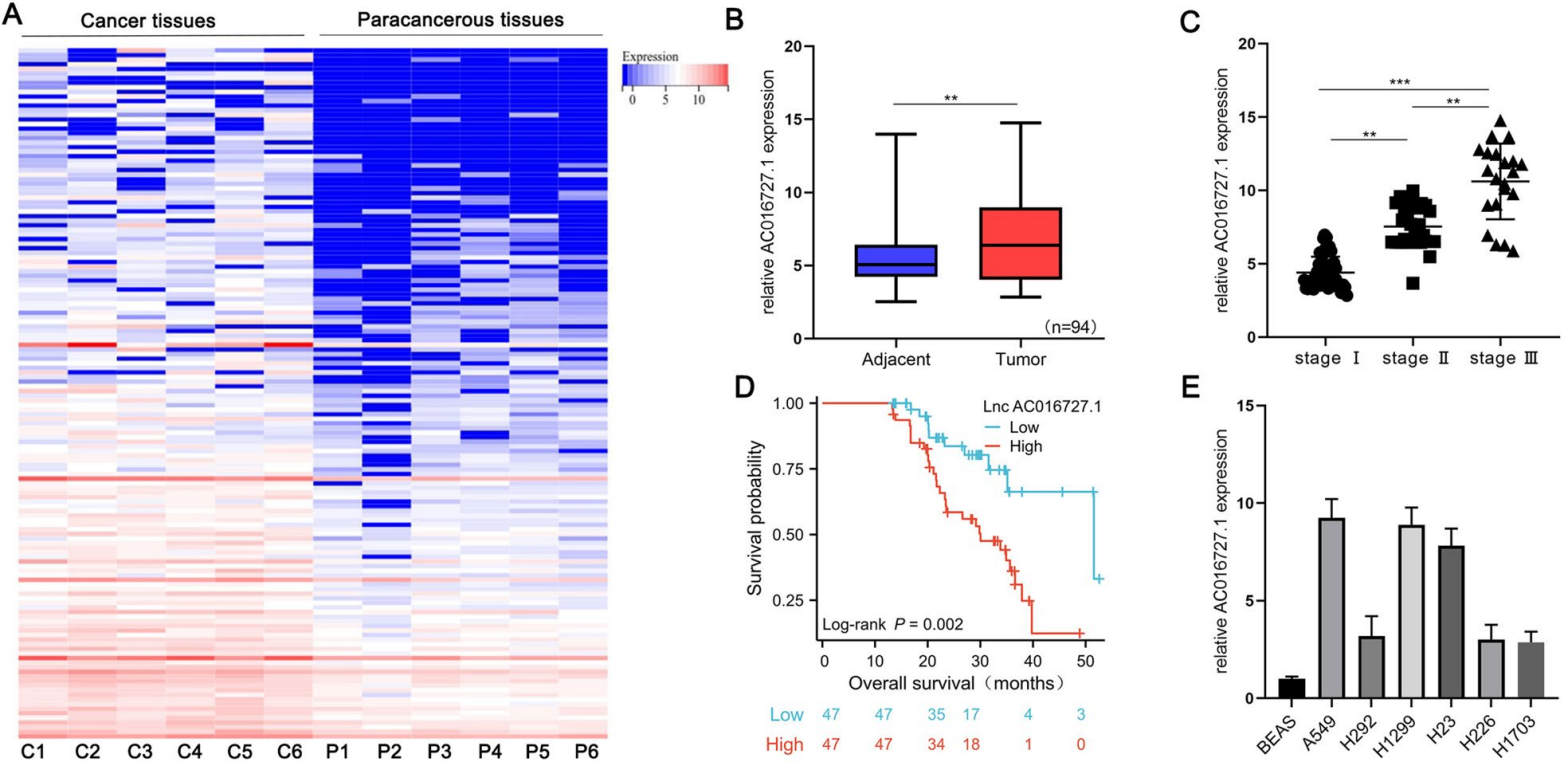
Clinical significance of lncRNA AC016727.1 upregulation in NSCLC tissues and different NSCLC cell subtypes. **a** Heatmap showing the six lncRNAs pairs upregulated in NSCLC tumor tissues in comparison to paired paracancerous tissues. **b** Relative expression of lncRNA AC016727.1 in NSCLC and paired paraneoplastic tissues (*n* = 94). **c** Relative expression of lncRNA AC016727.1 at different TNM stages in patients with NSCLC. **d** Kaplan–Meier analysis showing that lncRNA AC016727.1 expression in patients with NSCLC is associated with the overall survival of patients. **e** Comparing the relative expression of lncRNA AC016727.1 in several NSCLC cell subtypes to that of normal cells (*p < 0.05, **p < 0.01, and ***p < 0.001)

We conducted transcriptome resequencing on NSCLC and matched paracancerous tissues under strict quality control conditions to determine whether lncRNAs participate in the progression of NSCLC. Following screening criteria (fold change ≥ 1.5, *p*-value < 0.05, false discovery rate < 0.05), we identified that lncRNA AC016727.1 was significantly upregulated in NSCLC tissues (Fig. 1A). We performed RT-qPCR on 94 pairs of NSCLC and their paired paracancerous tissues to determine the clinical significance of lncRNA AC016727.1 expression. Our findings showed that lncRNA AC016727.1 expression increased significantly in tumor tissues and that this increasing trend was associated with the progression of the TNM stage (*p* < 0.05, Fig. 1B and C). Then, to investigate the clinical importance of lncRNA AC016727.1 expression in NSCLC tissues, we divided patients into two groups. Table 1 demonstrates a significant correlation between high lncRNA AC016727.1 expression and lymph node metastasis (*p* = 0.03), TNM stage (*p* = 0.008), tumor stage (*p* = 0.008), and tumor size (*p* = 0.002) among patients with NSCLC. However, lncRNA AC016727.1 did not show any significant associations with age, sex, distant metastasis, or tumor multiplicity. Additionally, univariate analysis demonstrated that lymph node metastasis, TNM stage, and tumor grade were all correlated with overall survival. Both lncRNA AC016727.1 expression (*p* = 0.03) and TNM stage (*p* = 0.04) were found to serve as significant independent predictors of prognosis for patients with NSCLC, based on the outcomes of Cox proportional hazard regression analysis (Table 2). High expression of lncRNA AC016727.1 was associated with significantly poorer overall survival in a Kaplan–Meier analysis of the 94 patients with NSCLC (*p* = 0.002, Fig. 1D). High lncRNA AC016727.1 expression was linked to poor overall survival (*p* = 0.02, Supplementary Fig. 1A) and progression-free interval (*p* = 0.01, Supplementary Fig. 1B), according to a combined analysis of genotype-tissue expression data and data from the Cancer Genome Atlas Program. These findings collectively imply that the lncRNA AC016727.1 expression level is elevated in patients with NSCLC and is linked to both poor clinical features and prognosis.

### lncRNA AC016727.1 promotes NSCLC growth in vivo and in vitro

**Fig. 2:**
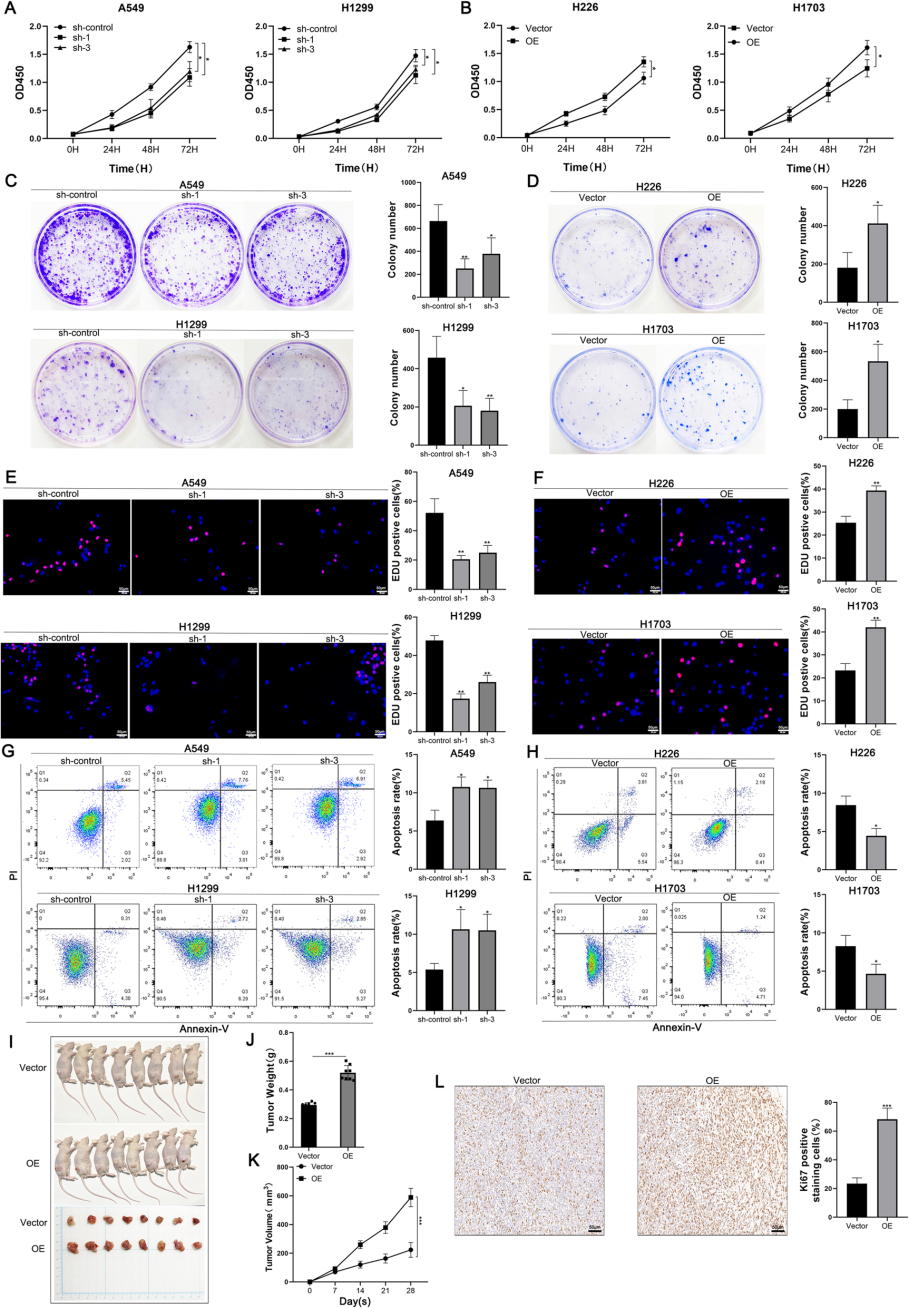
In in vivo settings, lncRNA AC016727.1 stimulates the growth of many NSCLC cell subtypes and tumorigenesis.** a** The CCK-8 assay showed that lncRNA AC016727.1 knockdown decreased A549 and H1299 cell proliferation. **b** The CCK-8 experiment showed that overexpression of lncRNA AC016727.1 increased H226 and H1703 cell proliferation. **c-d** In the colony formation assay, lncRNA AC016727.1 affects A549, H1299, H226, and H1703 cell proliferation. Quantitative analysis is presented on the right. **e–f** Effect of lncRNA AC016727.1 on the DNA synthesis activity of A549, H1299, H226, and H1703 cells via the EdU assay. Quantitative analysis is presented on the right. **g-h** Flow cytometry analysis of the effect of lncRNA AC016727.1 on apoptosis in A549, H1299, H226, and H1703 cells. **i-k** Cell lines stably overexpressing lncRNA AC016727.1 were subcutaneously injected into nude mice. The effect of lncRNA AC016727.1 overexpression on tumor size, weight, and volume. **l** IHC staining and quantitative analysis of Ki67 in xenograft tumors (***p < 0.001, **p < 0.01, and *p < 0.05)

To elucidate the biological functions of lncRNA AC016727.1 in NSCLC cells, we measured its expression in BEAS-2B and various lung cancer cell lines, including A549, H1299, H292, H23, H226, and H1703, RT-qPCR. The expression of lncRNA AC016727.1 exhibited significant heterogeneity among different lung cancer cell lines (Fig. 1E). Subsequently, we investigated the effects of lentiviral-mediated shRNA knockdown of lncRNA AC016727.1 on A549, H1299, and H23 cells, which exhibited higher lncRNA AC016727.1 expression (Supplementary Fig. 1C). Conversely, we overexpressed lncRNA AC016727.1 in H226 and H1703 cells, where its expression was relatively low (Supplementary Fig. 1D). The findings of the EdU, colony formation, and CCK-8 assays showed that lncRNA AC016727.1 knockdown remarkably reduced cellular activity and proliferation in H1299, H23, and A549 cells (*p* < 0.05, Fig. 2A, C, E, and Supplementary Fig. 2A, B, C). Conversely, overexpression of lncRNA AC016727.1 was observed to increase cell proliferation in H226 and H1703 cells (*p* < 0.05, Fig. 2B, D, and F). Additionally, lncRNA AC016727.1 knockdown increased the overall apoptosis rate in H1299, H23, and A549 cells, according to flow cytometry data (*p* < 0.05, Fig. 2G and Supplementary Fig. 2D), whereas lncRNA AC016727.1 overexpression significantly reduced the overall apoptosis rate in H226 and H1703 cells (*p* < 0.05, Fig. 2H). Furthermore, we developed xenograft mouse models by injecting treated A549 cells subcutaneously, revealing that lncRNA AC016727.1 overexpression resulted in significant increases in tumor weight and volume (*p* < 0.05, Figure 2I, J, K). Subsequently, we conducted an IHC analysis of tumors formed by A549 cells, which revealed that Ki67 staining, indicative of cell proliferation capacity, was significantly increased after lncRNA AC016727.1 overexpression in comparison to the vector group (*p* < 0.05, Fig. 2L).

### lncRNA AC016727.1 promotes the migration and EMT process of NSCLC cells in vitro and in vivo

**Fig. 3:**
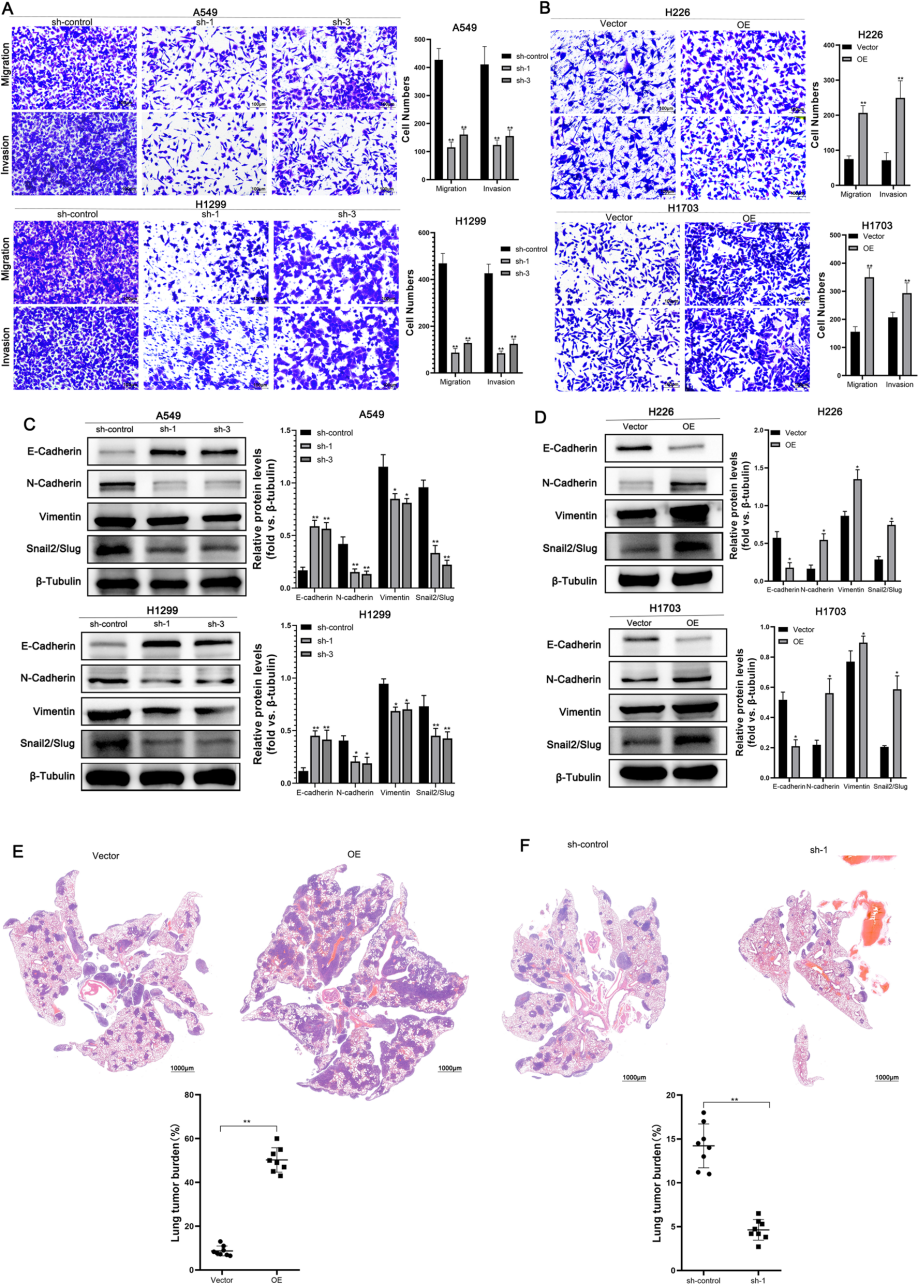
lncRNA AC016727.1 enhances cell migration and EMT. **a-b** Effect of lncRNA AC016727.1 on A549, H1299, H226, and H1703 cells' ability to migrate and invade as determined using the Transwell assay. On the right, quantitative analysis is displayed. **c-d** Expression of EMT marker proteins in different NSCLC cell subtypes following lncRNA AC016727.1 knockdown or overexpression. **e–f** Cell lines overexpressing or knocking down lncRNA AC016727.1 were injected into nude mice's tail veins. HE staining and quantitative analysis of xenograft tumor lung metastatic nodules by number (***p < 0.001, **p < 0.01, and *p < 0.05)

We employed a Transwell system to examine the effects of lncRNA AC016727.1 on the migration and invasion of NSCLC cell lines. Knockdown of lncRNA AC016727.1 led to a significant decrease in the invasiveness of A549, H1299, and H23 cells (*p* < 0.05, Fig. 3A and Supplementary Fig. 2E). Similarly, overexpression of lncRNA AC016727.1 significantly enhanced the migration and invasion abilities of H226 and H1703 cells (*p* < 0.05, Fig. 3B). In cancer, the EMT is associated with tumor initiation, invasion, and metastasis [17]. Therefore, we further explored whether lncRNA AC016727.1 participates in the EMT process in NSCLC. Western blot analysis revealed that knockdown of lncRNA AC016727.1 upregulated E-cadherin (the epithelial marker) and downregulated the N-cadherin, vimentin, and Snail2/Slug (mesenchymal markers) in A549, H1299, and H23 cells (*p* < 0.05, Fig. 3C and Supplementary Fig. 2F). Moreover, overexpression of lncRNA AC016727.1 was shown to promote the EMT process in H226 and H1703 cells (*p* < 0.05, Fig. 3D).

In addition, we injected A549 cells with lncRNA AC016727.1 overexpression into Balb/c nude mice to examine its effect on lung cancer metastasis in vivo. Histological examination revealed that a high level of lncRNA AC016727.1 promoted the metastasis of A549 cells to the lungs (Fig. 3E), confirming its role in promoting the growth and metastasis of NSCLC.

To further confirm these findings, we conducted experiments using LLC cells, a mouse NSCLC cell line. We observed elevated expression of lncRNA AC016727.1 in LLC compared to mouse normal alveolar cells (Supplementary Fig. 5A). The clone formation assay demonstrated increased cell proliferation after overexpression of lncRNA AC016727.1 in LLC cells (*p* < 0.05, Supplementary Fig. 5B). Furthermore, the Transwell assay indicated that both invasive and migratory abilities of cells were increased after lncRNA AC016727.1 overexpression (*p* < 0.05, Supplementary Fig. 5C). These results were consistent with the orthotopic lung tumor model (Supplementary Fig. 5D).

### lncRNA AC016727.1 sponges miR-98-5p in NSCLC cells

**Fig. 4:**
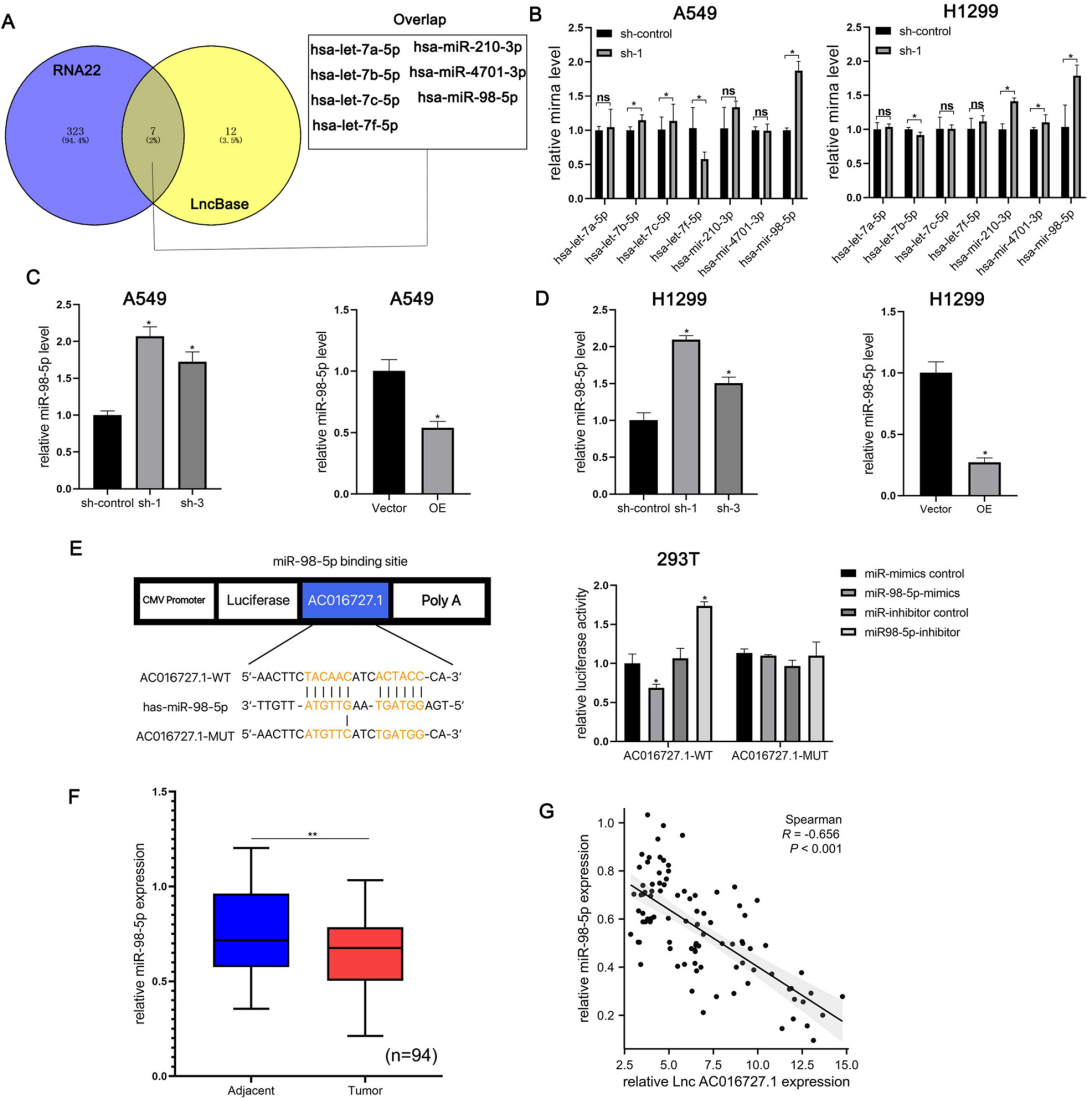
lncRNA AC016727.1 sponges miR-98-5p in different NSCLC cell subtypes.** a** Seven putative targets of lncRNA AC016727.1 were predicted using RNA22 and LncBase. **b-d** qRT-PCR shows that lncRNA AC016727.1 downregulates miR-98-5p in A549 and H1299 cells. **e** Sequence alignment of miR-98-5p with the putative binding sites of lncRNA AC016727.1. Binding of lncRNA AC016727.1 and miR-98-5p was verified using the luciferase reporter assay.** f** Relative expression of miR-98-5p in NSCLC and paired paraneoplastic tissues (*n* = 94). **g** Correlation between the expression of lncRNA AC016727.1 and miR-98-5p in NSCLC tissues (*n* = 94(***p < 0.001, **p < 0.01, and *p < 0.05)

One of the key pathways via which lncRNAs exert their effects is through competing endogenous mechanisms at the transcriptional or post-transcriptional level. We hypothesized that lncRNA AC016727.1 may function through its ceRNA mechanism. To explore this, we used two online bioinformatics tools, RNA22 (https://cm.jefferson.edu/rna22/Interactive/) and LncBase (https://diana.e-ce.uth.gr/lncbasev3/home), to predict potential target miRNAs for lncRNA AC016727.1. Among the predicted miRNAs, seven miRNAs overlapped between these two prediction lists (hsa-miR-98-5p, hsa-let-7a-5p, hsa-let-7b-5p, hsa-let-7c-5p, hsa-let-7f-5p, hsa-miR-210-3p, and hsa-miR-4701-3p) (Fig. 4A). To determine which miRNAs are primarily regulated by lncRNA AC016727.1, we examined the effect of lncRNA AC016727.1 depletion on the levels of seven predicted miRNAs using RT-qPCR. Among the seven miRNAs examined, lncRNA AC016727.1 was found to negatively regulate miR-98-5p in both H1299 and A549 cells (Fig. 4B, C, and D).

According to research, downregulated miR-98-5p is closely linked to the onset and progression of a variety of different malignancies (*e.g*., pancreatic ductal adenocarcinoma [18], gastric cancer [19], bladder cancer [20], and other cancers [21, 22]). We therefore conducted a dual luciferase reporter test to ascertain if lncRNA AC016727.1 and miR-98-5p directly interact. miR-98-5p significantly and negatively regulated the activity of luciferase in the wt lncRNA AC016727.1 vector, but not in the mut vector, according to dual luciferase reporter analyses (*p* < 0.5, Fig. 4E). These findings show that miR-98-5p can bind to the lncRNA AC016727.1 directly. The levels of miR-98-5p were then assessed in paired tissue samples. As expected, NSCLC tissues downregulated miR-98-5p (*p* < 0.05, Fig. 4F), and miR-98-5p exhibited a negative association with the expression of the lncRNA AC016727.1 (*r* =  − 0.656, *p* < 0.0001, Fig. 4G). These data imply that in NSCLC cell lines, lncRNA AC016727.1 works as a molecular sponge for miR-98-5p and that its biological function is mediated by miR-98-5p.

**Fig. 5:**
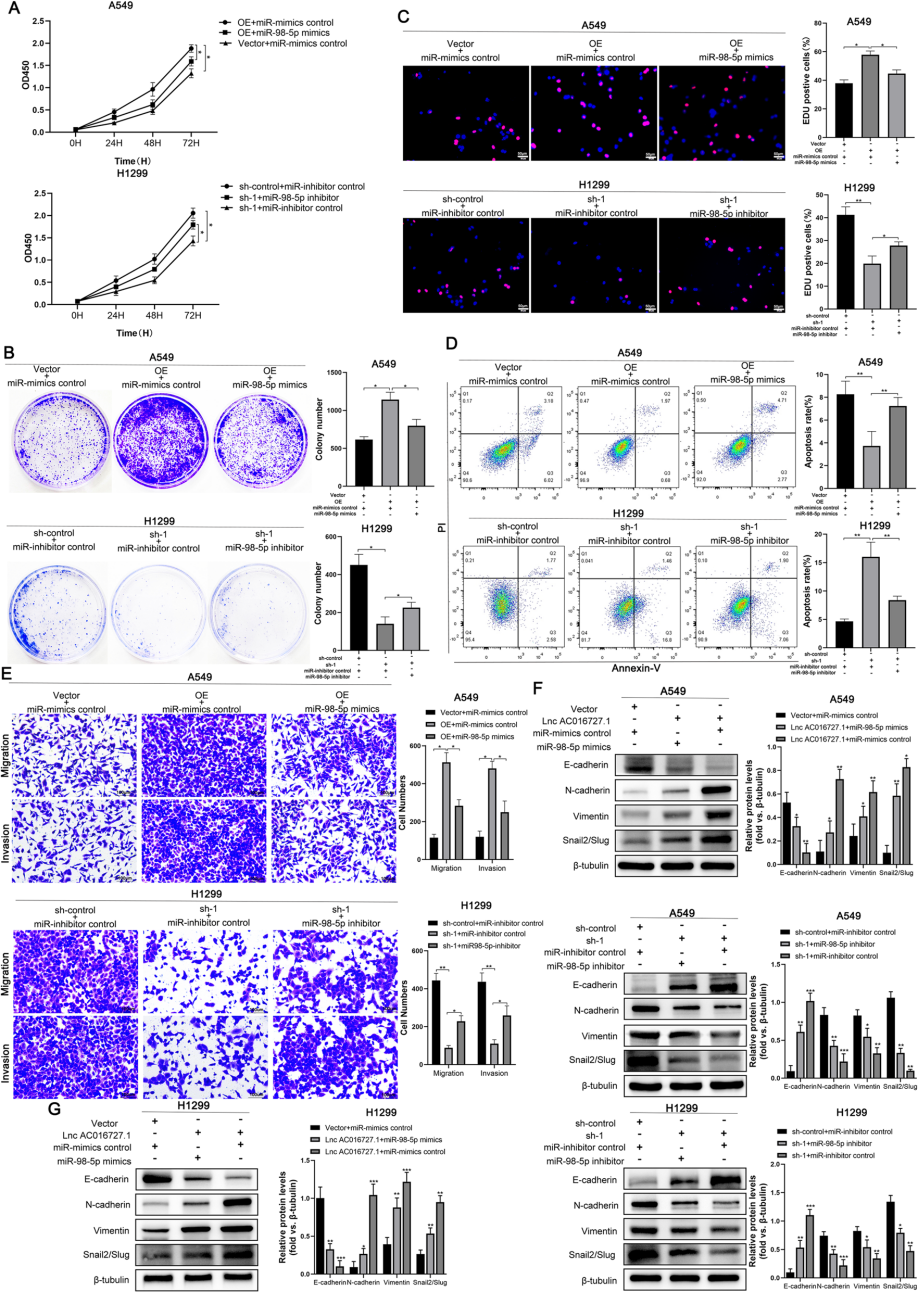
Biological function of lncRNA AC016727.1 is mediated by miR-98-5p. **a-c** miR-98-5p mimics inhibited the proliferation of lncRNA AC016727.1-overexpressing A549 cells. The miR-98-5p inhibitor promoted the proliferation of H1229 cells after lncRNA AC016727.1 knockdown. Quantitative analysis is presented on the right. **d** miR-98-5p decreased apoptosis in A549 cells overexpressing the lncRNA AC016727.1. After lncRNA AC016727.1 suppression, the miR-98-5p inhibitor increased H1229 cell proliferation. Quantitative analysis is presented on the right. **e** miR-98-5p mimics attenuated the migratory and invasive properties of A549 cells overexpressing the lncRNA AC016727.1. After lncRNA AC016727.1 knockdown, the miR-98-5p inhibitor increased the migratory and invasive capacities of H1229 cells. Quantitative analysis is presented on the right. **f-g**. miR-98-5p mimics inhibited the expression of EMT marker proteins in lncRNA AC016727.1-overexpressing A549 cells. The miR-98-5p inhibitor increased H1229 cell EMT marker protein expression after lncRNA AC016727.1 suppression. Quantitative analysis is presented on the right (***p < 0.001, **p < 0.01, and *p < 0.05)

To further elucidate whether miR-98-5p mediates the biological function of lncRNA AC016727.1, we conducted rescue experiments. We found that miR-98-5p mimics effectively counteracted the pro-proliferative effect of lncRNA AC016727.1 overexpression in A549 cells using the CCK-8, colony formation, and EdU assays. Conversely, the miR-98-5p inhibitors partially reversed the anti-proliferative effects triggered by lncRNA AC016727.1 knockdown in H1299 cells (*p* < 0.5, Fig. 5A, B, C). Furthermore, flow cytometry analysis revealed that co-transfection with miR-98-5p mimics in A549 cells resisted the inhibition of apoptosis caused by lncRNA AC016727.1 overexpression, whereas miR-98-5p inhibitors countered the overall decrease in apoptosis resulting from lncRNA AC016727.1 knockdown in H1299 cells (*p* < 0.5, Fig. 5D). Subsequently, migration and invasion assays demonstrated that in A549 cells, miR-98-5p mimics resisted the enhancement of cell invasion and migration abilities induced by lncRNA AC016727.1 overexpression. Conversely, inhibiting miR-98-5p expression in H1299 cells restrained the increased cell invasion and migration capacities caused by lncRNA AC016727.1 (*p* < 0.05, Fig. 5E). Similarly, we established that the EMT progression, which was inhibited by lncRNA AC016727.1 knockdown and promoted by its overexpression, could be partially restored by miR-98-5p inhibitors and mimics, as demonstrated by western blot assays (*p* < 0.05, Fig. 5F and G). These rescue experiments provide evidence that lncRNA AC016727.1 functions as a molecular sponge for miR-98-5p in NSCLC cells and exerts its biological effects by sequestering miR-98-5p in NSCLC cells.

### miR-98-5p targets BACH1

**Fig. 6:**
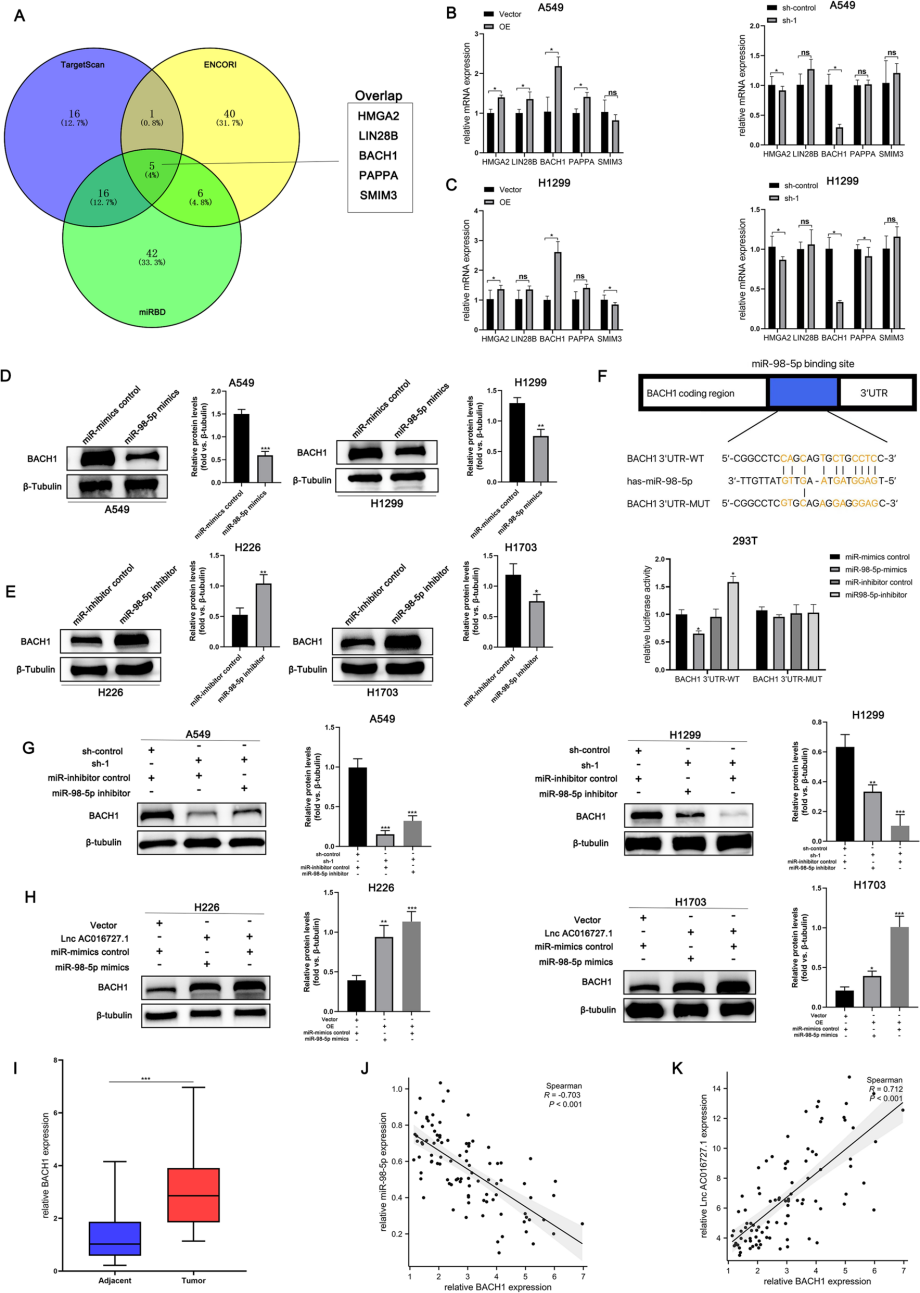
miR-98-5p targets BACH1.** a** Five potential miR-98-5p targets were predicted using TargetScan, ENCORI, and miRDB. **b-c** Five possible targets' mRNA expression in A549 and H1299 cells following overexpression or knockdown of the lncRNA AC016727.1. **d-e** Effect of miR-98-5p on BACH1 protein levels.** f** Sequence alignment of miR-98-5p with BACH1. Binding of miR-98-5p and BACH1 was verified using the per-luciferase reporter assay. **g-h** The miR-98-5p inhibitor inhibited BACH1 expression in A549 and H1299 cells after lncRNA AC016727.1 knockdown. miR-98-5p mimics increased BACH1 expression in H226 and H173 cells overexpressing the lncRNA AC016727.1. Quantitative analysis is presented on the right. **i** Relative BACH1 expression in NSCLC and paired paraneoplastic tissues (*n* = 94). **j** Relative correlation between the expression of miR-98-5p and BACH1 in NSCLC tissues (*n* = 94). **k** Relative correlation between the expression of lncRNA AC016727.1 and BACH1 in NSCLC tissues (*n* = 94) (***p < 0.001, **p < 0.01, and *p < 0.05)

miRNAs regulate the expression of their target genes. Three bioinformatics databases, namely, miRDB (https://www.mirdb.org/), TargetScan (https://www.targetscan.org/vert_80/), and ENCORI (https://starbase.sysu.edu.cn/index.php) were utilized to predict the possible targets of miR-98-5p. Figure 6A illustrates that five potential target genes, i.e., HMGA2, LIN28B, BACH1, PAPPA, and SMIM3, overlap with each other in the predictions using the three databases. lncRNA AC016727.1 was overexpressed and knocked down in A549 and H1299 cell lines, respectively, followed by RT-qPCR analysis. We observed that only BACH1 correlated with lncRNA AC016727.1 expression in the overexpression or knockdown groups in comparison to the normal group (Fig. 6B and C). Furthermore, we observed a strong correlation between BACH1 and NSCLC development and progression.

In contrast, using ENCORI, we observed a significant negative correlation between miR-98-5p and BACH1 expression in NSCLC (*r* =  − 0.097, *p* < 0.05, Supplementary Fig. 3). To further confirm this finding, A549, H1299, and H23 cells were transfected with miR-98-5p mimics. miR-98-5p mimics significantly inhibited BACH1 protein levels after cell transfection (*p* < 0.05, Fig. 6D and Supplementary Fig. 2G). On the other hand, BACH1 protein levels significantly increased in miR-98-5p inhibitor-transfected H226 and H1703 cells compared to controls (*p* < 0.05, Fig. 6E). These findings point to miR-98-5p exerting a negative regulatory influence on BACH1 expression in NSCLC cells. We then tested whether miR-98-5p and BACH1 mRNA can interact directly. The luciferase reporter assay demonstrated that miR-98-5p mimics or inhibitors affected the luciferase activity of wild-type BACH1 vectors in a significant and negative manner; however, they had no effects on the activity of mutant vectors (*p* < 0.05 Fig. 6F). Furthermore, Western blotting demonstrated that miR-98-5p inhibitors may partially restore the drop in lncRNA AC016727.1 knockdown-induced BACH1 protein levels following the transfection of A549, H1299, and H23 cells (*p* < 0.05, Fig. 6G and Supplementary Fig. 2H). On the other hand, lncRNA AC016727.1 partially inhibited the inhibitory effect of miR-98-5p mimics on BACH1 protein levels in H226 and H1703 cells (*p* < 0.05, Fig. 6H). BACH1 expression level was also higher in NSCLC tissues than in non-tumor tissues (*p* < 0.0001, Fig. 6I). According to statistical analysis, BACH1 expression was negatively linked to miR-98-5p expression (*r* =  − 0.703, *p* < 0.001, Fig. 6J) but positively correlated with lncRNA AC016727.1 expression (*r* = 0.712, *p* < 0.001, Fig. 6K). Collectively, these data and experimental results suggest that lncRNA AC016727.1 positively alters BACH1 expression by inhibiting miR-98-5p.

### lncRNA AC016727.1 promotes tumor proliferation, invasive migration, and aerobic glycolytic progression via BACH1

Abnormal energy metabolism, particularly the oncogenic glycolysis regulation and the multifaceted role of glycolytic components, is a major feature of cancer and highlights the biological significance of tumor glycolysis [23]. In lung cancer, the BACH1 protein can promote glycolytic metabolism, intracellular glucose uptake, and lactate excretion [24, 25]. Furthermore, in breast cancer, BACH1 can inhibit the expression of electron transport chain-related genes [25, 26]. Therefore, we explored whether lncRNA AC016727.1 participates in aerobic glycolysis in NSCLC. The protein levels of several glucose transporter proteins and metabolic enzymes were measured. Western blotting revealed that lncRNA AC016727.1 knockdown downregulated glycolytic proteins in A549, H1299, and H23 cells, including HK2, MCT1, and phosphofructokinase-2/fructose-2,6-bisphosphatase 3 (PFKFB3) (*p* < 0.05, Supplementary Fig. 4A). In contrast, lncRNA AC016727.1 overexpression promoted aerobic glycolysis in H226 and H1703 cells (*p* < 0.5, Supplementary Fig. 4B).

**Fig. 7:**
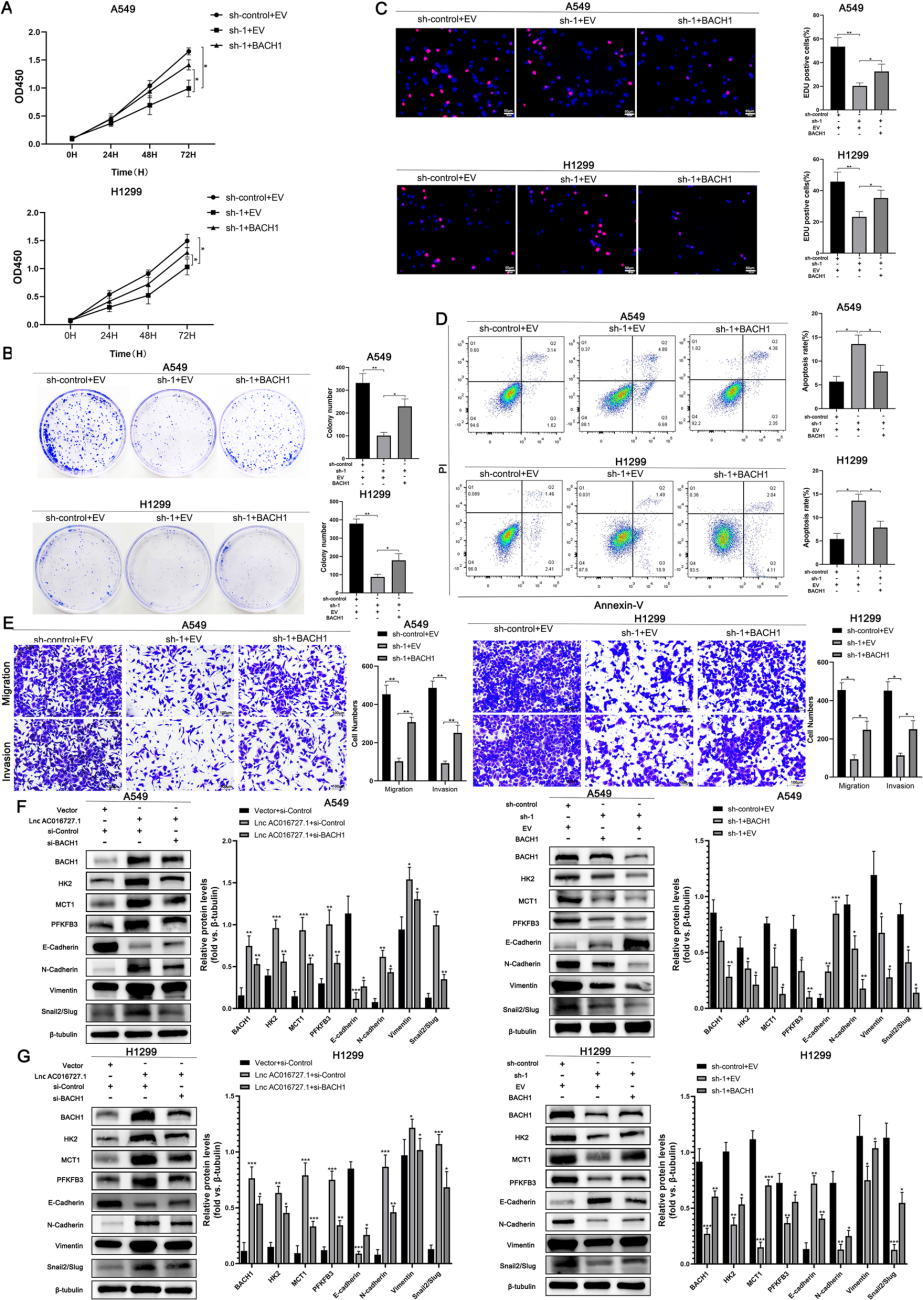
lncRNA AC016727.1 promotes tumor proliferation, invasive migration, and aerobic glycolytic progression via BACH1. **a-c** BACH1 enhanced A549 and H1299 proliferation following lncRNA AC016727.1 knockdown. Quantitative analysis is presented on the right. **d** BACH1 inhibited A549 and H1299 cell apoptosis following lncRNA AC016727.1 knockdown. Quantitative analysis is presented on the right. **e** BACH1 promoted the migratory and invasive capacities of lncRNA AC016727.1-overexpressing A549 and H1299 cells. Quantitative analysis is presented on the right. **f** Following overexpression or knockdown of the lncRNA AC016727.1, EMT and cellular glycolysis marker proteins were identified to assess the impact of BACH1 on EMT in the A549 cells. Quantitative analysis is presented on the right. **g** Following overexpression or silencing of the lncRNA AC016727.1, EMT and cellular glycolysis marker proteins were detected to assess the impact of BACH1 on EMT in the H1299 cells. Quantitative analysis is presented on the right

We also observed that lncRNA AC016727.1 upregulated BACH1 levels by sponging miR-98-5p. We performed rescue experiments to determine whether the biological functions of lncRNA AC016727.1 are mediated via BACH1. The EdU, colony formation, and CCK-8 assays revealed that BACH1 overexpression in A549 and H1299 cells partially abrogated cell proliferation effects of lncRNA AC016727.1 knockdown (*p* < 0.05, Fig. 7A, B, C). Meanwhile, flow cytometry revealed that lncRNA AC016727.1 knockdown-induced apoptosis was partially eliminated after BACH1 overexpression (*p* < 0.05, Fig. 7D). Next, the Transwell assay revealed that after lncRNA AC016727.1 knockdown, BACH1 increased A549 and H1299 cells' capacity for migration and invasion (*p* < 0.05, Fig. 7E). Moreover, glucose uptake and lactate production assays revealed that after lncRNA AC016727.1 knockdown, lactate synthesis, and glucose uptake were significantly decreased in H1299 and A549 cells; however, these phenomena were reversed by BACH1 overexpression (*p* < 0.05, Supplementary Fig. 4C and D). The effect of lncRNA AC016727.1 on glucose metabolism was then determined using the Seahorse XF Cell Mito Stress and Glycolysis Stress Test kits, which are standard methodologies for reflecting the flux distribution of glucose metabolism. lncRNA AC016727.1 knockdown increased maximal respiration in the OCR assay (*p* < 0.05, Supplementary Fig. 4E and F). However, BACH1 alleviated this increase. On the other hand, the ECAR assay revealed that lncRNA AC016727.1 knockdown decreased the glycolytic capacity of A549 and H1299 cells (*p* < 0.05, Supplementary Fig. 4E and F). Furthermore, this assay revealed that lncRNA AC016727.1 knockdown decreased the glycolytic capacity of A549 and H1299 cells; however, BACH1 overexpression partially reversed this decrease in glycolytic capacity (*p* < 0.05, Supplementary Fig. 4E and F). Similarly, we clarified that miR-98-5p inhibitors and mimics promoted and impended the glycolytic capacity, which was partially restored by BACH1 (*p* < 0.05, Supplementary Fig. 4G and H). Furthermore, western blotting revealed that lncRNA AC016727.1 knockdown and overexpression affected A549 and H1299 cells' EMT-related protein production, which was partially restored by BACH1 overexpression and knockdown. Similarly, the decrease in the levels of glycolysis-related proteins HK2, MCT1, and PFKFB3 in A549 and H1299 cells caused by lncRNA AC016727.1 knockdown was partially restored by BACH1 and vice versa (*p* < 0.05, Fig. 7F and G). Collectively, these findings suggest that lncRNA AC016727.1 promotes tumor proliferation, aggressive migration, and aerobic glycolytic progression via BACH1.

### Hypoxia regulates the lncRNA AC016727.1/BACH1/HIF-1α signaling loop

**Fig. 8:**
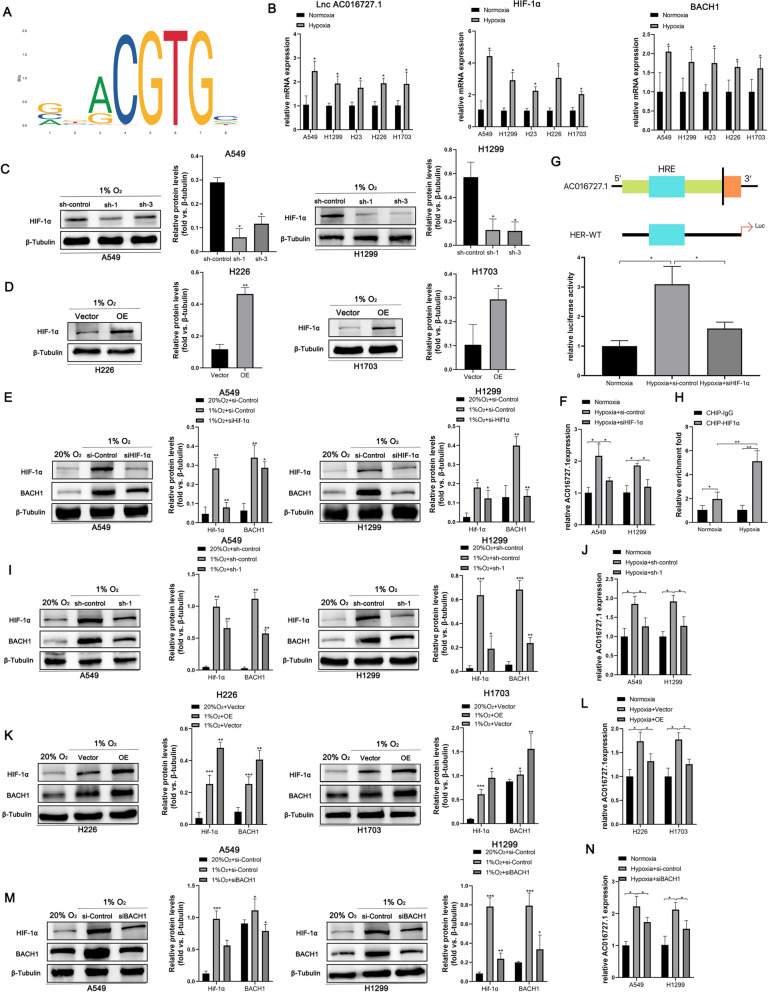
Hypoxia regulates the lncRNA AC016727.1/BACH1/HIF-1α signaling loop. **a** The recognition interaction motif of HIF-1α and lncRNA A016727.1 was predicted using the JSAPAR database.** b** mRNA expression of lncRNA AC016727.1, HIF-1α, and BACH1 in five different NSCLC cell subtypes (A549, H1299, H23, H266, and H1703 cells) under normoxic and hypoxic conditions. **c** HIF-1α expression was inhibited in A549 and H1299 cells following lncRNA AC016727.1 knockdown under aerobic conditions. Quantitative analysis is presented on the right. **d** HIF-1α expression was promoted in H226 and H1703 cells after lncRNA AC016727.1 overexpression under aerobic conditions. On the right, quantitative analysis is shown. **e–f** Expression of HIF-1α, BACH1, and lncRNA AC016727.1 after HIF-1α knockdown in A549 and H1299 cells under normoxic and hypoxic conditions. **g** Hypoxia-sensing elements in the lncRNA AC016727.1 promoter. Binding of lncRNA AC016727.1 and HIF-1α was validated using the luciferase reporter assay. **h** Under normoxic and hypoxic conditions, a ChIP assay was performed to verify the binding between the HRE of the lncRNA AC016727.1 promoter and HIF-1ɑ. **i**-**j** Expression of HIF-1α, BACH1, and lncRNA AC016727.1 after lncRNA AC016727.1 knockdown in A549 and H1299 cells under normoxic and hypoxic conditions. **k**-**l** Expression of HIF-1α, BACH1, and lncRNA AC016727.1 after lncRNA AC016727.1 overexpression in H226 and H1703 cells under normoxic and hypoxic conditions. **m–n** Expression of HIF-1α, BACH1, and lncRNA AC016727.1 following BACH1 knockdown in H226 and H1703 cells under normoxic and hypoxic conditions

**Fig. 9:**
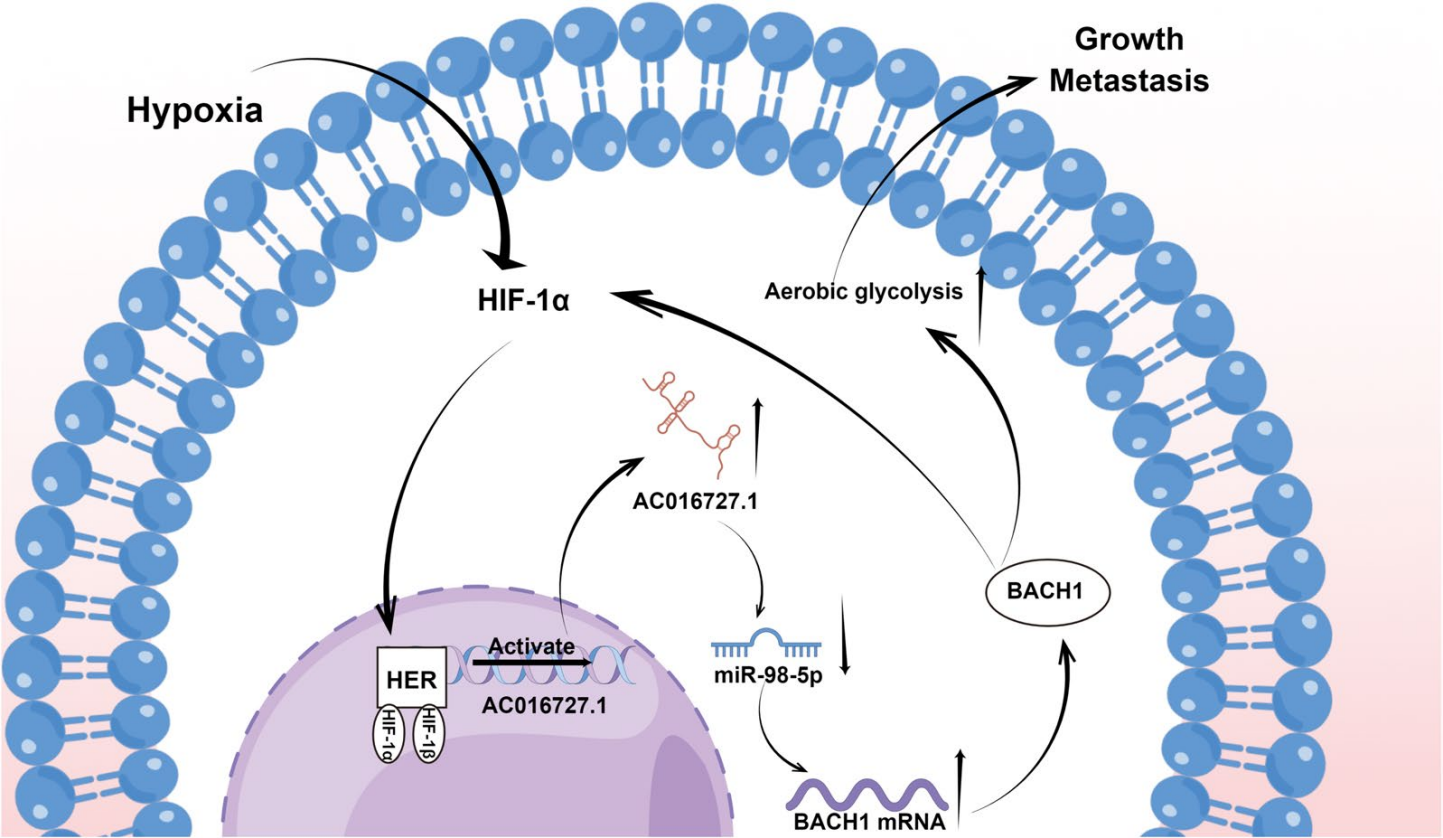
Schematic illustration of the mechanism via which the lncRNA AC016727.1/BACH1/HIF-1 α signaling loop promotes NSCLC progression

According to recent research, hypoxia in the tumor microenvironment is related to a poor patient prognosis. Hypoxia is one of the primary reasons for treatment resistance and poor survival in patients with NSCLC [27]. Subsequently, the JASPAR web tool indicated a possible hypoxia response element (HRE) in lncRNA AC016727.1 (Fig. 8A). We wished to elucidate whether hypoxic conditions affect lncRNA AC016727.1 expression. Therefore, we cultured A549, H1299, H23, H226, and H1703 cells. RT-qPCR indicated that the expression of lncRNA AC016727.1, HIF-1α, and BACH1 was differentially increased in all five cell lines under hypoxia conditions compared to normoxic conditions. (*p* < 0.05, Fig. 8B). Next, western blotting findings showed a considerable drop in HIF-1α levels in A549, H1299, and H23 under hypoxic conditions with lncRNA AC016727.1 knockdown in comparison to normoxic conditions (*p* < 0.05, Fig. 8C and Supplementary Fig. 2I). In contrast, HIF-1α expression was significantly increased in lncRNA AC016727.1-overexpressing H226 and H1703 cells compared to controls (*p* < 0.05, Fig. 8D). Collectively, these findings indicated a possible interaction between HIF-1α and lncRNA AC016727.1. We selected H1299 and A549 cells transfected with short interfering RNAs against HIF-1α. Western blotting revealed that HIF-1α downregulation significantly decreased hypoxia-induced lncRNA AC016727.1 and BACH1 upregulation (*p* < 0.05, Fig. 8E and F). This further confirms that HIF-1α may regulate hypoxia-induced lncRNA AC016727.1 and BACH1 expression. The luciferase assay demonstrated that hypoxia significantly increased lncRNA AC016727.1 promoter vector-transfected cells' luciferase activity and that HIF-1α knockdown reversed hypoxia-induced luciferase activity (*p* < 0.05, Fig. 8G). Additionally, the ChIP assay demonstrated direct HIF-1 and HRE binding in the promoter of the lncRNA AC016727.1(*p* < 0.05, Fig. 8H). Subsequently, western blotting revealed that hypoxia-induced HIF-1α and BACH1 upregulation was significantly attenuated after low lncRNA AC016727.1 expression (*p* < 0.05, Fig. 8I and J) and vice versa (*p* < 0.05, Figure 8K and L). Finally, the effect of BACH1 on HIF-1α and lncRNA AC016727.1 was investigated. We observed that HIF-1α and lncRNA AC016727.1 levels were increased in a hypoxic environment. Furthermore, BACH1 downregulation partially reversed this change (*p* < 0.05, Fig. 8M and N). Collectively, our findings suggest that HIF-1α can affect lncRNA AC016727.1 expression and that lncRNA AC016727.1 regulates HIF-1α expression via BACH1, thereby forming a hypoxia-mediated lncRNA AC016727.1/BACH1/HIF-1α signaling loop in NSCLC (Fig. 9).

## Discussion

Recent research has achieved major advances in comprehending the molecular mechanisms that underlie lung cancer development; yet, the prognosis for individuals with advanced lung cancer remains dismal. lncRNAs are aberrantly expressed in different tumor types and may be novel biomarkers and therapeutic targets for cancer [28]. Studies have reported that several lncRNAs are associated with NSCLC progression in various ways [29–31], providing additional perspectives on understanding NSCLC occurrence and progression. ACC016727.1 was found as a new lncRNA that is upregulated in NSCLC tissues using whole transcriptome sequencing. It may be closely associated with lung carcinogenesis and progression.

Functionally, lncRNA A016727.1 enhances NSCLC cell growth and metastasis in vivo and in vitro, as well as their glycolytic capacity. For the first time, we discovered the carcinogenic role of lncRNA AC016727.1 in NSCLC in this study. Next, we identified a novel mechanism in which lncRNA AC016727.1 regulates BACH1. We discovered that BACH1 may be a target of miR-98-5p in NSCLC by combining bioinformatics research and experimental validation. In NSCLC cells, miR-98-5p was negatively correlated with BACH1 expression; however, lncRNA AC016727.1 expression was positively correlated with BACH1. In addition, by performing a series of rescue experiments, we demonstrated that the oncogenic function of lncRNA AC016727.1 was mediated by BACH1 in NSCLC cells. Therefore, our study findings suggest that lncRNA AC016727.1 can promote BACH1 expression in NSCLC by affecting miR-98-5p.

Hypoxia is a microenvironmental feature of cancer and is closely associated with cancer progression. Many studies have demonstrated the involvement of lncRNAs via HIF-1α regulation in the effects of hypoxic conditions on tumor biological behavior. The lncRNA PVT1, for example, modulates nasopharyngeal carcinoma cell proliferation by stabilizing HIF-1α and activating KAT2A acetyltransferase [32]. Additionally, the regulatory loop formed by the lncRNA HITT and HIF-1 regulates angiogenesis and tumor progression [33]. In the present investigation, we determined for the first time that the lncRNA AC016727.1 is hypoxia-sensitive. Experimentally, we found that hypoxia induced the expression of the lncRNA AC016727.1; however, HIF-1ɑ downregulation inhibited this expression. Additionally, we identified a putative HRE in the promoter of the lncRNA AC016727.1 using the JASPAR database. The possible regulatory role of HIF-1α in the transcription of lncRNA AC016727.1 was validated using the dual luciferase reporter assay. Moreover, we observed that the upregulation of HIF-1α and BACH1 expression under hypoxic conditions was attenuated by lncRNA AC016727.1 knockdown and that BACH1 knockdown reversed the increase in HIF-1α and lncRNA AC016727.1 expression under hypoxic conditions.

These findings imply that HIF-1α is a transcription factor that activates lncRNA AC016727.1 transcription and that lncRNA AC016727.1 regulates HIF-1α expression via BACH1, thereby forming the lncRNA AC016727.1/BACH1/HIF-1α signaling loop in hypoxic environments.

## Conclusions

In NSCLC, we found that hypoxia-induced lncRNA AC016727.1 is a promoter of tumors and is closely related to tumor progression. Through the miR-98-5p/BACH1 axis, lncRNA AC016727.1 enhances the growth and metastasis of NSCLC cells. Furthermore, as a transcription factor, HIF-1α can activate lncRNA AC016727.1 transcription. lncRNA AC016727.1 may regulate HIF-1α expression via BACH1, thereby forming the lncRNA AC016727.1/BACH1/HIF-1α signaling loop in a hypoxic environment. Our findings elucidate the functions of lncRNAs in NSCLC progression and identify a potential therapeutic target and predictor for NSCLC.

### Supplementary Information

**Additional file 1:** **Table S1.** Primers used in the study.

**Additional file 2: Table S2.** Antibodies used in the study.

**Additional file 3:** **Supplementary Fig.** 1 a-b Kaplan–Meier analysis showing that high lncRNA AC016727.1 expression is correlated with overall survival (OS) and progression-free interval (PFI) in patients with NSCLC. c-d Efficiency of the virus transfection of A549, H1299, H23, H266, and H1703 cells.

**Additional file 4:** **Supplementary Fig. 2.** a lncRNA AC016727.1 knockdown inhibited the proliferation of H23 cells via the CCK-8 assay. b Effect of lncRNA AC016727.1 on the proliferation of H23 cells via the colony formation assay. Quantitative analysis results are presented on the right. c Effect of lncRNA AC016727.1 on the DNA synthesis activity of H23 cells via the EdU assay. Quantitative analysis is presented on the right. d Effect of lncRNA AC016727.1 on the apoptosis of H23 cells via flow cytometry. e Effect of lncRNA AC016727.1 on H23 cell migration and invasion using Transwell assay. Quantitative analysis is presented on the right. f Expression of EMT marker proteins in H23 cells following lncRNA AC016727.1 knockdown or overexpression. g Effect of miR-98-5p on levels of BACH1 protein. h Western blot analysis confirmed that lncRNA AC016727.1 and miR-98-5p can interact to regulate the expression of BACH1. I Western blotting showing HIF-1α expression following lncRNA AC016727.1 knockdown in H23 cells. j-k Effect of BACH1 on the expression of HIF-1ɑ and lncRNA AC016727.1 under hypoxic conditions.

**Additional file 5:** **Supplementary Fig. 3.** Pan-cancer analysis to determine the correlation between miR-98-5p and lncRNA AC016727.1 expression in tumor tissues collected from patients with NSCLC.

**Additional file 6:** **Supplementary Fig. 4.** lncRNA AC016727.1 promotes tumor proliferation, aggressive migration, and aerobic glycolytic progression via BACH1. a HK2, MCT1 and PFKFB expression as inhibited in A549, H1299, and H23 cells following lncRNA AC016727.1 knockdown. Quantitative analysis is presented on the right. b HK2, MCT1, and PFKFB expression was promoted in H226 and H23 cells after lncRNA AC016727.1 overexpression. Quantitative analysis results are presented on the right. c-d After lncRNA AC016727.1 knockdown, BACH1 enhanced glucose absorption and lactate generation in A549 and H1299 cells. e-f Effects of BACH1 on glycolysis in A549 and H1299 cells through assessing OCR and ECAR following lncRNA AC016727.1 knockdown. The black arrows indicate the time point at which the cells were processed. Quantitative analysis results are presented on the right. g-h Effects of miR-98-5p on glycolysis in A549 and H1299 cells through assessing OCR and ECAR following BACH1 overexpression or knockdown. The time point at which the cells were treated is indicated by the black arrows. Quantitative analysis is presented on the right (*p < 0.05, **p < 0.01, and ***p < 0.001).

**Additional file 7:** **Supplementary Fig.** 5lncRNA AC016727.1 promotes the proliferation, migration, and invasion of mouse non-small cell lung carcinoma cells, Lewis lung carcinoma (LLC), and tumorigenesis in vivo. a Relative expression of lncRNA AC016727.1 in LLC in comparison to normal cells. b Effect of lncRNA AC016727.1 on the proliferation of LLC cells via the colony formation assay. c Effect of lncRNA AC016727.1 on LLC cell’s migration and invasion capacities using Transwell assay. d Effect of lncRNA AC016727.1 overexpression on tumors in the orthotopic lung tumor model.

## Data Availability

The datasets supporting the conclusions of this article are included within the manuscript.

## Abbreviations

ceRNA: Competing endogenous RNA
ECAR: Extracellular acidification rate
HR: Hazard ratio
IHC: Immunohistochemistry
lncRNA: Long noncoding RNA
NSCLC: Non-small cell lung cancer
OCR: Oxygen consumption rate
OS: Overall survival
PFI: Progression-free interval
SCLC: Small cell lung cancer
TNM: Tumor node metastasis staging system
