# Supplementary material for: Lnc AC016727.1/BACH1/HIF-1 α signal loop promotes the progression of non-small cell lung cancer
Source: J Exp Clin Cancer Res. 2023 Nov 10;42:296. doi: 10.1186/s13046-023-02875-y (PMC10636976; doi:10.1186/s13046-023-02875-y)
Supplement: Supplementary file 1 — Additional file 1: Table S1. Primers used in the study. [file 13046_2023_2875_MOESM1_ESM.docx]

**Table S1：primers used in the study**

| Gene | Primer sequences (5’-3’) |
| --- | --- |
| Lnc AC016727.1-F(human) | CCGCTCTCCCCATAACTCAA |
| Lnc AC016727.1-R（human） | TCATTTGCCTGCCAATCCAA |
| BACH1-F | AAATGAATGCCTGGGAGGAGT |
| BACH1-R | AATTGTGGGGGAAAGGAGTCA |
| miR-98-5p-F | GCGCCGTGAGGTAGTAAGTTGTATTGTT |
| let-7a-5p-F | GCGCGTGAGGTAGTAGGTTGTATAGTT |
| let-7b-5p-F | GGTGGTGAGGTAGTAGGTTGTGTGGTT |
| let-7c-5p-F | GGTAGGTGAGGTAGTAGGTTGTATGGTT |
| let-7f-5p-F | GCGGTGCTGAGGTAGTAGATTGTATAGTT |
| miR-210-3p-F | CTGTGCGTGTGACAGCGGCT |
| miR-4701-3p-F | ATGGGTGATGGGTGTGGTGT |
| U6-F | GCTTCGGCAGCACATATACTAAAAT |
| HIF-1α-F | ACCCTAACTAGCCGAGGAAGA |
| HIF-1α-R | ACCAAGCAGGTCATAGGTGG |
| β-ACTIN-F | CTCCATCCTGGCCTCGCTGT |
| β-ACTIN-R | GCTGTCACCTTCACCGTTCC |
| HMGA1-F | CCAGTGAAGTGCCAACTCCGAAG |
| HMGA1-R | GCCCTCCTCTTCCTCCTTCTCC |
| LIN28B-F | CCAGCCATGCACTTCAACTCTCC |
| LIN28B-R | TGACCTGCCTGACCGTTCTGAG |
| PAPPA-F | TCTGCTGGACACGAGTCTGGAG |
| PAPPA-R | CACCTTGGGCTGGCGGAAAC |
| SMIM3-F | GCAGTCAGCCAAGTCCCCATG |
| SMIM3-R | GCCAGGATGATGAGGACAATAACCC |
| Lnc AC016727.1-HER-F | GTGGAGTTCATACACATGAGGGA |
| Lnc AC016727.1-HER-R | GGGGAACAGGTGGTTTTTGG |
| Lnc AC016727.1-F(mouse) | AGGAGTGGGTGAGGGATAAGACTAC |
| Lnc AC016727.1-R（mouse） | GCACCTGTCATGTGAGCAGTATG |
