# Supplementary material for: Lnc AC016727.1/BACH1/HIF-1 α signal loop promotes the progression of non-small cell lung cancer
Source: J Exp Clin Cancer Res. 2023 Nov 10;42:296. doi: 10.1186/s13046-023-02875-y (PMC10636976; doi:10.1186/s13046-023-02875-y)
Supplement: Supplementary file 2 — Additional file 2: Table S2. Antibodies used in the study. [file 13046_2023_2875_MOESM2_ESM.docx]

| Antibody | Source | Catalog |
| --- | --- | --- |
| E-Cadherin | Proteintech | Cat#20874-1-AP |
| N-Cadherin | Proteintech | Cat#22018-1-AP |
| Vimentin | Proteintech | Cat#10366-1-AP |
| BACH1 | Proteintech | Cat#14018-1-AP |
| HK2 | Proteintech | Cat#22029-1-AP |
| PFKFB3 | Proteintech | Cat#13763-1-AP |
| MCT1 | Proteintech | Cat#20139-1-AP |
| HIF-1α | Proteintech | Cat#20960-1-AP |
| β-Actin | Proteintech | Cat#20536-1-AP |
| Ki-67 | Proteintech | Cat#27309-1-AP |
| Snail2/Slug | Proteintech | Cat#12129-1-AP |

**Table S2. Antibodies used in the study**
