# Supplementary figures and images for: Lnc AC016727.1/BACH1/HIF-1 α signal loop promotes the progression of non-small cell lung cancer
Source: J Exp Clin Cancer Res. 2023 Nov 10;42:296. doi: 10.1186/s13046-023-02875-y (PMC10636976; doi:10.1186/s13046-023-02875-y)

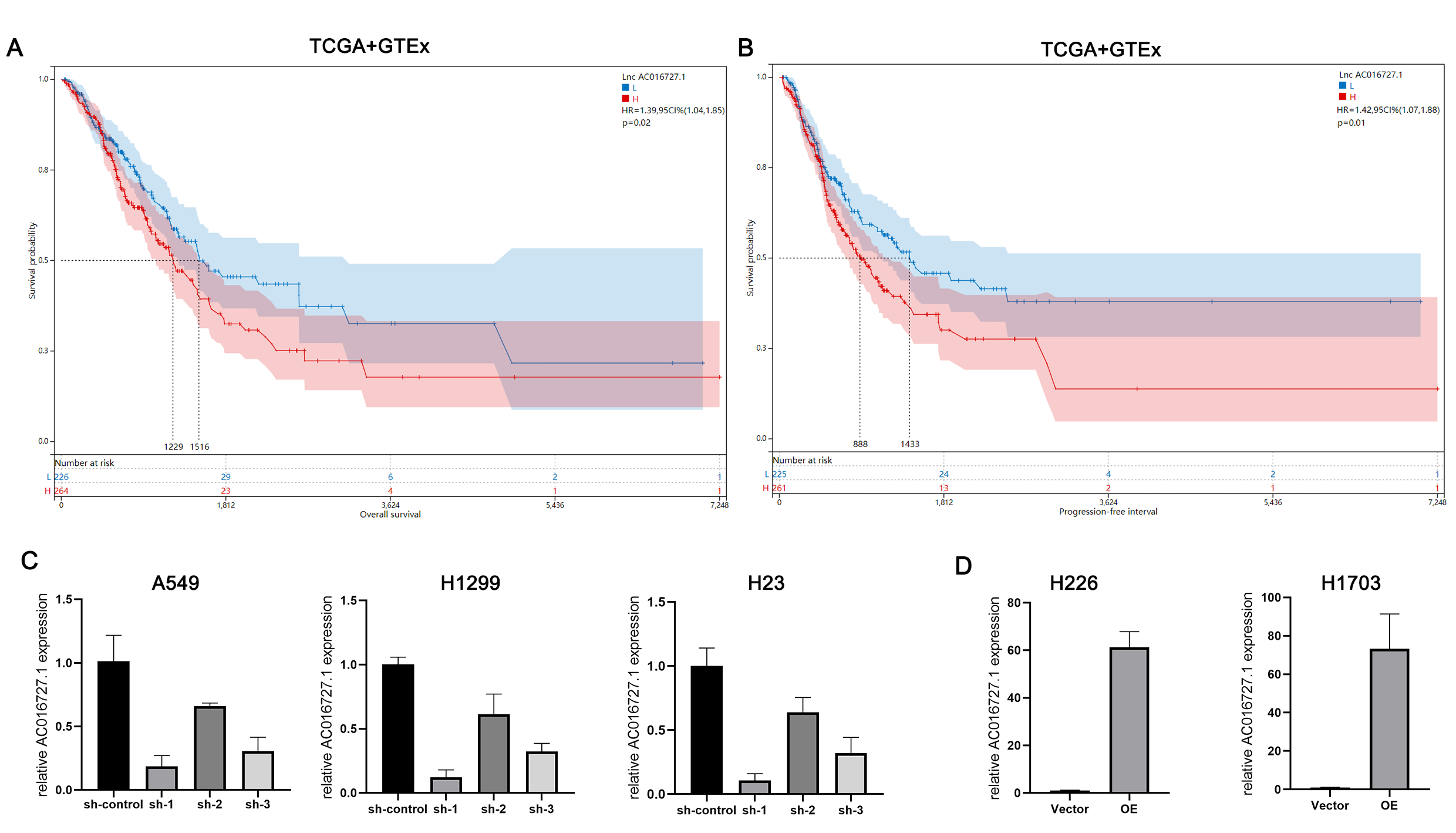

Supplement: Supplementary file 3 — Additional file 3: Supplementary Fig. 1 a-b Kaplan–Meier analysis showing that high lncRNA AC016727.1 expression is correlated with overall survival (OS) and progression-free interval (PFI) in patients with NSCLC. c-d Efficiency of the virus transfection of A549, H1299, H23, H266, and H1703 cells. [file 13046_2023_2875_MOESM3_ESM.tif]

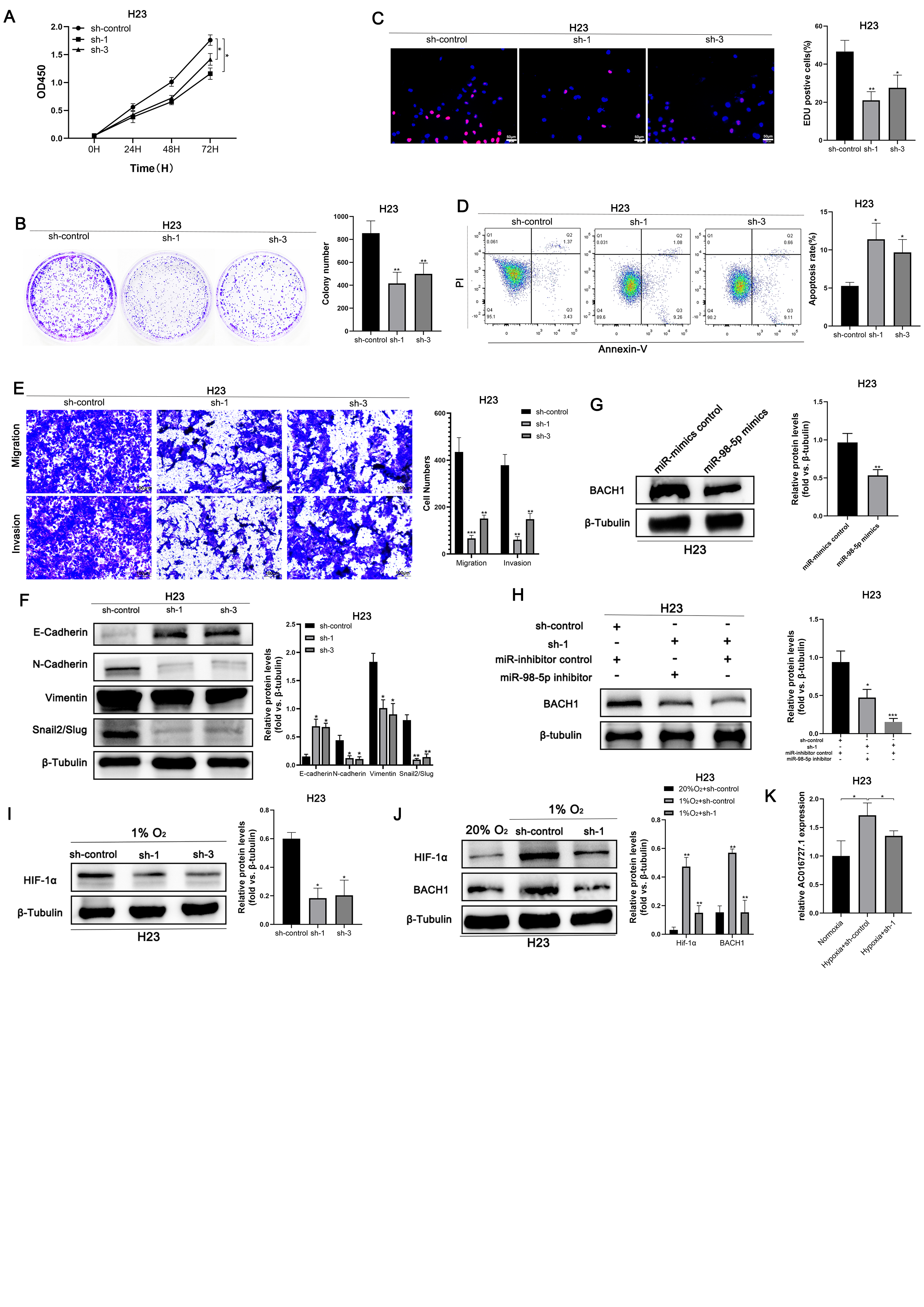

Supplement: Supplementary file 4 — Additional file 4: Supplementary Fig. 2. a lncRNA AC016727.1 knockdown inhibited the proliferation of H23 cells via the CCK-8 assay. b Effect of lncRNA AC016727.1 on the proliferation of H23 cells via the colony formation assay. Quantitative analysis results are presented on the right. c Effect of lncRNA AC016727.1 on the DNA synthesis activity of H23 cells via the EdU assay. Quantitative analysis is presented on the right. d Effect of lncRNA AC016727.1 on the apoptosis of H23 cells via flow cytometry. e Effect of lncRNA AC016727.1 on H23 cell migration and invasion using Transwell assay. Quantitative analysis is presented on the right. f Expression of EMT marker proteins in H23 cells following lncRNA AC016727.1 knockdown or overexpression. g Effect of miR-98-5p on levels of BACH1 protein. h Western blot analysis confirmed that lncRNA AC016727.1 and miR-98-5p can interact to regulate the expression of BACH1. I Western blotting showing HIF-1α expression following lncRNA AC016727.1 knockdown in H23 cells. j-k Effect of BACH1 on the expression of HIF-1ɑ and lncRNA AC016727.1 under hypoxic conditions. [file 13046_2023_2875_MOESM4_ESM.tif]

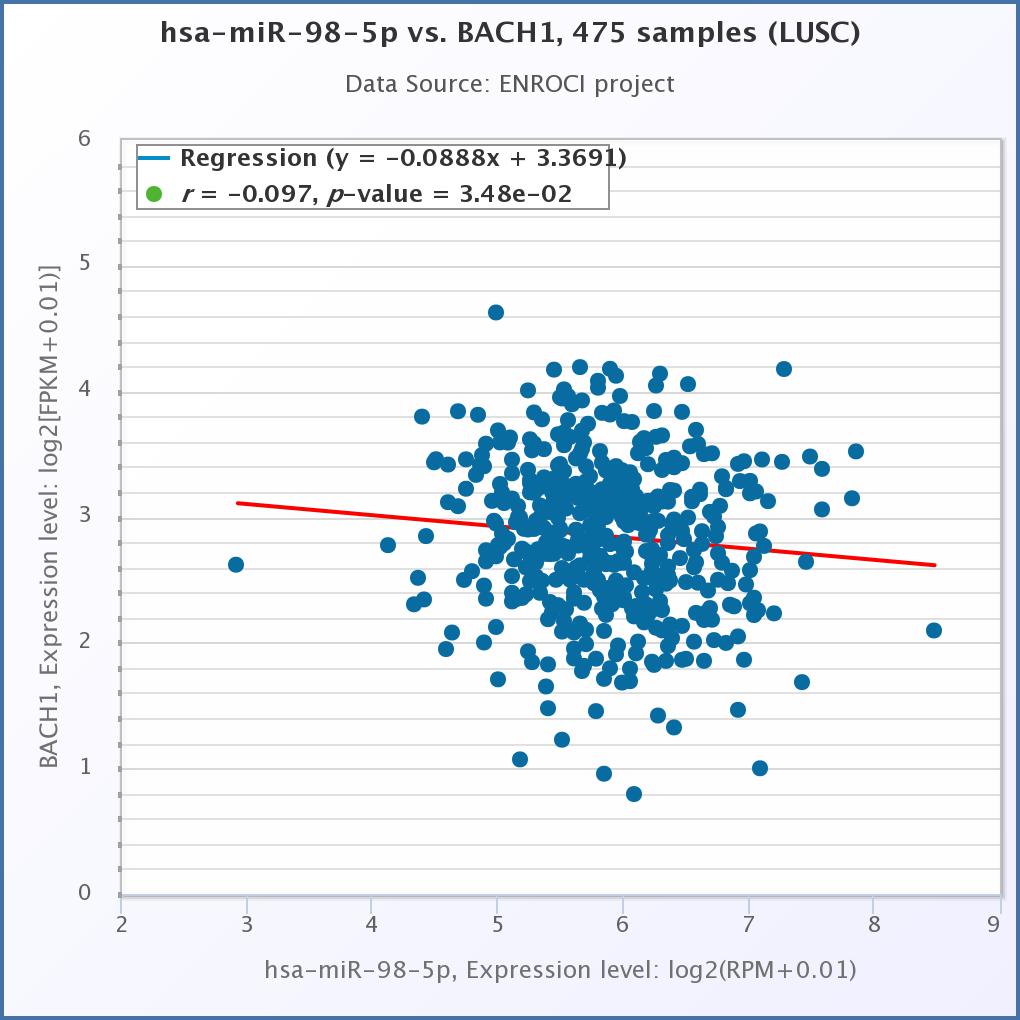

Supplement: Supplementary file 5 — Additional file 5: Supplementary Fig. 3. Pan-cancer analysis to determine the correlation between miR-98-5p and lncRNA AC016727.1 expression in tumor tissues collected from patients with NSCLC. [file 13046_2023_2875_MOESM5_ESM.jpeg]

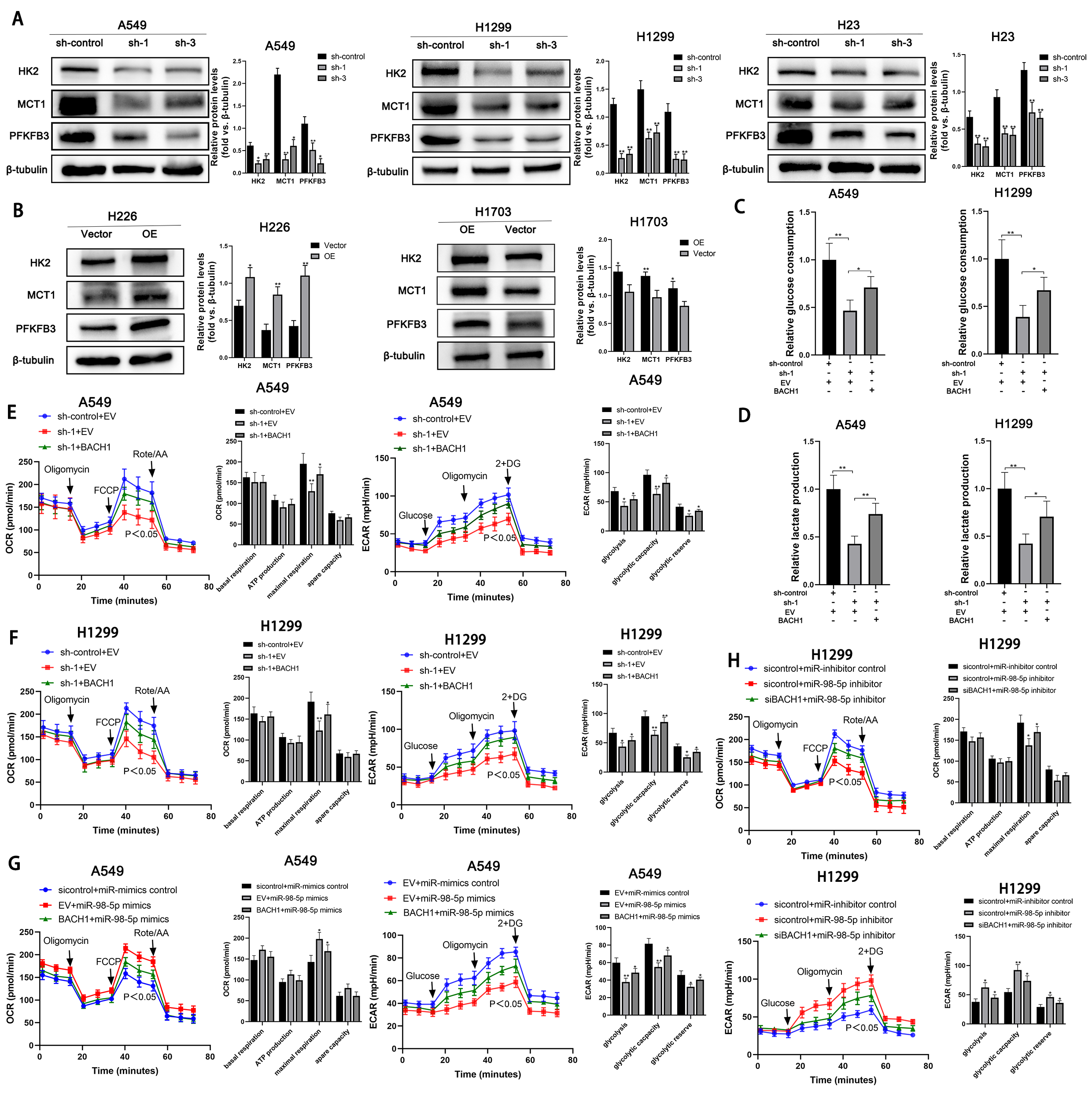

Supplement: Supplementary file 6 — Additional file 6: Supplementary Fig. 4. lncRNA AC016727.1 promotes tumor proliferation, aggressive migration, and aerobic glycolytic progression via BACH1. a HK2, MCT1 and PFKFB expression as inhibited in A549, H1299, and H23 cells following lncRNA AC016727.1 knockdown. Quantitative analysis is presented on the right. b HK2, MCT1, and PFKFB expression was promoted in H226 and H23 cells after lncRNA AC016727.1 overexpression. Quantitative analysis results are presented on the right. c-d After lncRNA AC016727.1 knockdown, BACH1 enhanced glucose absorption and lactate generation in A549 and H1299 cells. e-f Effects of BACH1 on glycolysis in A549 and H1299 cells through assessing OCR and ECAR following lncRNA AC016727.1 knockdown. The black arrows indicate the time point at which the cells were processed. Quantitative analysis results are presented on the right. g-h Effects of miR-98-5p on glycolysis in A549 and H1299 cells through assessing OCR and ECAR following BACH1 overexpression or knockdown. The time point at which the cells were treated is indicated by the black arrows. Quantitative analysis is presented on the right (*p < 0.05, **p < 0.01, and ***p < 0.001). [file 13046_2023_2875_MOESM6_ESM.tif]

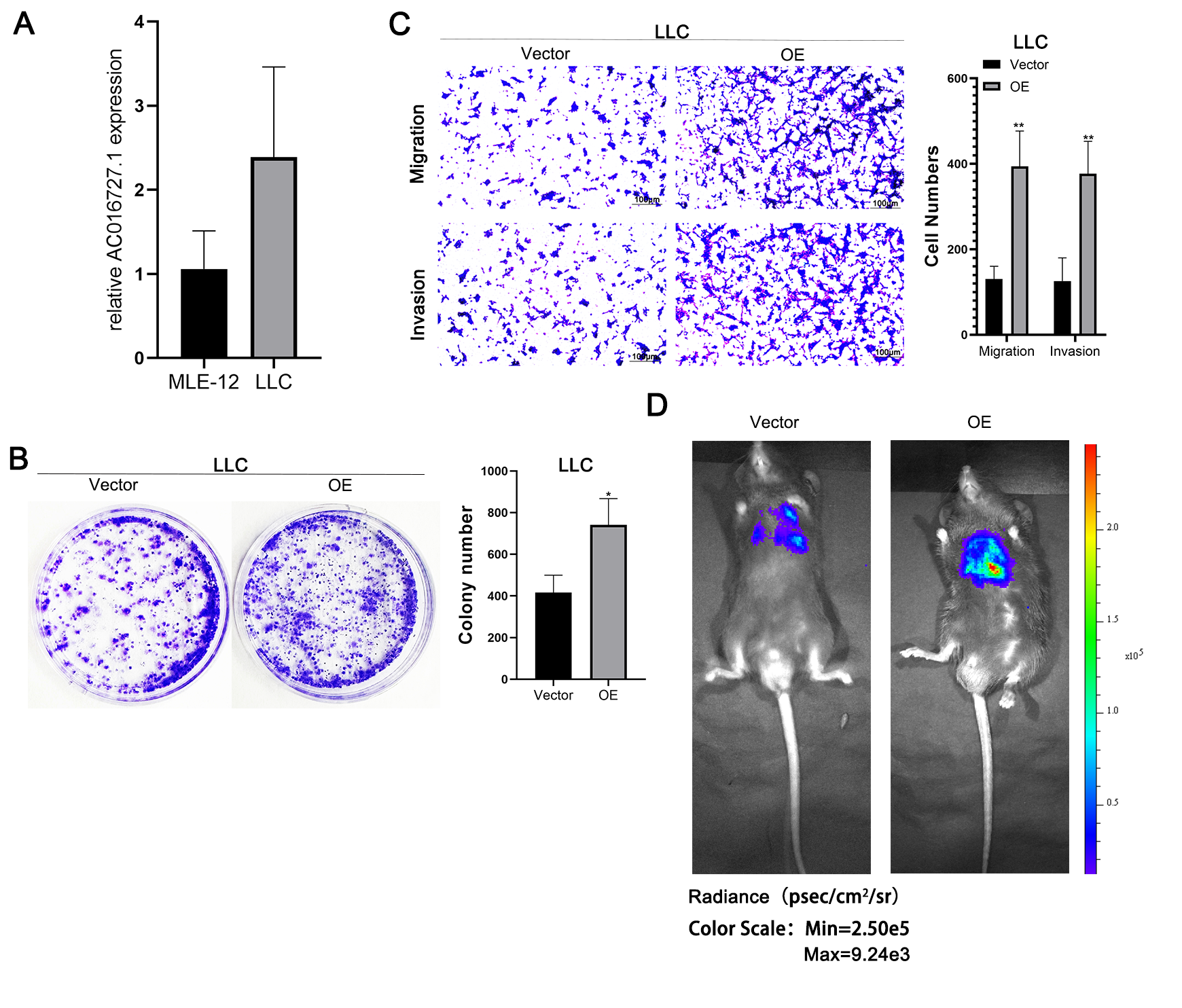

Supplement: Supplementary file 7 — Additional file 7: Supplementary Fig. 5lncRNA AC016727.1 promotes the proliferation, migration, and invasion of mouse non-small cell lung carcinoma cells, Lewis lung carcinoma (LLC), and tumorigenesis in vivo. a Relative expression of lncRNA AC016727.1 in LLC in comparison to normal cells. b Effect of lncRNA AC016727.1 on the proliferation of LLC cells via the colony formation assay. c Effect of lncRNA AC016727.1 on LLC cell’s migration and invasion capacities using Transwell assay. d Effect of lncRNA AC016727.1 overexpression on tumors in the orthotopic lung tumor model. [file 13046_2023_2875_MOESM7_ESM.tif]
